# Flocking propensity by satellites, but not core members of mixed-species flocks, increases when individuals experience energetic deficits in a poor-quality foraging habitat

**DOI:** 10.1371/journal.pone.0209680

**Published:** 2019-01-09

**Authors:** Katherine E. Gentry, Daniel P. Roche, Stephen G. Mugel, Nolan D. Lancaster, Kathryn E. Sieving, Todd M. Freeberg, Jeffrey R. Lucas

**Affiliations:** 1 Department of Biological Sciences, Purdue University, West Lafayette, Indiana, United States of America; 2 Department of Wildlife Ecology & Conservation, University of Florida, Gainesville, Florida, United States of America; 3 Department of Psychology, University of Tennessee, Knoxville, Tennessee, United States of America; 4 Department of Ecology and Evolutionary Biology, University of Tennessee, Knoxville, Tennessee, United States of America; Urbino University, ITALY

## Abstract

Mixed-species bird flocks are complex social systems comprising core and satellite members. Flocking species are sensitive to habitat disturbance, but we are only beginning to understand how species-specific responses to habitat disturbance affect interspecific associations in these flocks. Here we demonstrate the effects of human-induced habitat disturbance on flocking species’ behavior, demography, and individual condition within a remnant network of temperate deciduous forest patches in Indiana, USA. Specifically, we characterized the following properties of two core species, Carolina chickadees (*Poecile carolinensis*) and tufted titmice (*Baeolophus bicolor*), across a secondary-forest disturbance gradient: foraging time budgets, home range size, fat scores, fledgling counts, survival rates, and abundance. We also report fat scores for two satellite species that flock with the core study species: white-breasted nuthatches (*Sitta carolinensis*) and downy woodpeckers (*Dryobates pubescens*). Finally, we assess mixed-species flock sizes and composition, in addition to avian predator call rates, across the disturbance gradient. Foraging time budgets and home range size were highest and fat scores were lowest for core species in the most-disturbed site. Fat scores of two satellite species followed the same pattern. Additionally, the number of tufted titmice fledglings and winter survival rate of Carolina chickadee*s* were lowest at the most-disturbed site. These results suggest that core species in the most-disturbed site experienced energetic deficits. Moreover, cumulative calling rate of raptors was lowest at the most-disturbed site, and none of the individual raptor species call rates were higher at the most-disturbed site—suggesting that perception of predation risk does not contribute to these patterns. Surprisingly, the satellites continued associating with mixed species flocks through the breeding season at the most-disturbed site. Total flock size and interspecific association patterns were otherwise consistent across the gradient. The fact that satellites continued to flock with core species during the breeding season suggests foraging niche expansion resulting from mixed-species flocking is important in disturbed sites even beyond the winter season. Our study reveals mechanisms underlying flock composition of birds surviving in remnant forest and links the mechanisms to degradation of foraging habitat. These findings offer important insight into the relative impact potential of forest disturbance on mixed-species flocks in the North Temperate Zone.

## Introduction

Disturbance from human activities, including the implementation of forest management practices, can degrade the value of forest habitat by reducing the quantity, accessibility, and/or quality of key resources for wildlife communities [1]. The extent to which disturbance degrades habitat value depends on the resource requirements of individuals of each species [2], including food and resting habitat (e.g. [3]), and resources such as nesting sites that sustain intraspecific social organization (e.g.[4–6]). Forest degradation may also impact higher levels of social organization such as interspecific associations that, in turn, contribute to the composition and integrity of animal community structure ([7,8]). Mixed-species bird flocks represent a well-known example of these interspecific associations that are integral to bird community structure in forested biomes worldwide.

Mixed-species flocks typically contain both ‘core’ (or leading) and ‘satellite’ (or following) species seeking participation in facilitative relationships [9,10]. Core flocking species often act as attractants to satellite species [10] and all flock members can experience foraging or antipredator benefits (or both) by flocking together [11–13]. Indeed, the facilitative relationships that form from mixed-species flocking can extend the foraging niche space for participating members (see [14]); satellite species reduce their risk of predation [15,16] by attending to alarm calls and mobbing calls of the core species [17,18] and satellite species’ reliance on vocal signals from core species can enhance mobility through remnant forest networks [19–22]. Therefore, the effects of structural degradation of forest vegetation on core species can be indicative of community-wide responses among sympatric species, making core species key indicators of changes in habitat quality [7,15,23–25].

Flocking species are sensitive to habitat modification, and the response of core species to habitat disturbance can affect the long-term persistence of satellite species in disturbed habitat (see [7]). Not only are the foraging niches of satellite species dependent on the presence or absence of the core species [19,20], but the diminishment of core species in an area also makes satellite species particularly vulnerable to predation [26]. From a conservation perspective, therefore, it is especially informative to study how habitat disturbance affects core species; doing so can aid in defining landscape-scale best-management practices that potentially increase the species-holding capacity of remnant forests, and thus, biodiversity supported within fragmented landscapes [27,28]. Likewise, it is important to understand how the species in mixed-species groups differ in their sensitivities to disturbance, and how that variation subsequently affects the nature and composition of mixed-species groups and thus the relative value of interspecific associations [14].

Habitat disturbance effects are often evaluated using spatial distributions and population demographics such as survivorship [29,30], but these metrics provide only a coarse understanding of the susceptibility of a given population to habitat disturbance. A deeper understanding of habitat effects can be achieved by studying flocking dynamics across disturbance gradients. For example, assessment of interspecific associations in large, species-rich flocks revealed that the functional roles of flock members were not altered along a selective logging gradient in montane wet temperate forest [24]; in contrast, interspecific interactions in mixed-species flocks decayed across a different type of disturbance gradient within the Amazonian rainforest (primary forest, 100 ha fragments, 10 ha fragments, mixed primary and secondary forests, and secondary forest, [31]). Studies that consider finer scale measures of behavior and individual condition (e.g. time budgets, space use, and fat accumulation) in addition to distribution, demography, and flocking dynamics should provide even deeper insight into the impact of habitat gradients on flocking species.

We address the effects of forest disturbance on mixed-species bird flocks by describing how individual species in a low-diversity mixed-species flocking system respond to a secondary forest disturbance gradient within a remnant network of temperate deciduous forest. We characterized relevant aspects of the forest community at three sites that represent the disturbance gradient. We quantified behavioral, demographic, and physiological responses to the disturbance gradient in our core species through foraging time budgets, home range size, fat scores, fledgling counts, survival rates, and abundance. In addition, we characterized the mixed-species flock sizes and species composition across the disturbance gradient, as factors such as the abundance of flocking species, availability of resources, and intensity of predation risk can also affect the structural components of mixed-species flocks [8,23,27,32–35]). Collectively, our battery of measures allows us to assess a suite of mechanisms defining the impact potential of forest disturbance on flocking avifauna in the North Temperate Zone.

We hypothesized (1) that forest degradation (alteration of structure and composition) would alter habitat quality as reflected in individual, population, and flocking-level metrics selected to detect indicators of stressors at each level of organization. Specifically, we predicted that increased site disturbance would be associated with (individual level) expanded home range size, greater allocation of time spent foraging by core species, decreased fat loads, (population level) decreased abundance and fitness indices (survival, fledgling count). We also hypothesized that (2) the impact of disturbance on multiple behavioral and demographic traits in the core species would make them less attractive/effective as leaders to satellite species in disturbed habitat, in turn, degrading mixed species flock composition and size. Finally, (3) an important alternative hypothesis suggests that increased perception of predation risk can result in lower fat scores, reduced time spent foraging, and increased mixed-species flocking behavior. We tested the validity of this alternative hypothesis by measuring calling rates of predatory raptors at each of our sites.

## Methods

Data Availability Statement: All data and related metadata underlying the findings reported in this manuscript are deposited in the University of Florida public repository and can be accessed at http://ufdc.ufl.edu/l/IR00010449/00001/downloads.

### Study species

Our mixed species flocking system is composed of two core species: Carolina chickadees (*Poecile carolinensis*) and tufted titmice (*Baeolophus bicolor*). These species typically associate during winter in mixed-species flocks with two satellite species, white-breasted nuthatches (*Sitta carolinensis*) and downy woodpeckers (*Dryobates pubescens*). Carolina chickadees, hereafter CACH, and tufted titmice, hereafter TUTI, are resident songbirds from the family Paridae, and are characteristic of mature hardwood forests and late succession mixed hardwood-pine forests [36–39]. Where birds are generally well-known sentinels of habitat quality [40], those in the family Paridae are especially useful study subjects for assessing the consequences of habitat value as they readily occupy a range of habitats from undisturbed woodlands to fragmented and urban forests [41–43]. Downy woodpecker, hereafter DOWO (Family Picidae) and white-breasted nuthatch, hereafter WBNU (Family Sittidae), are the primary satellite species in mixed-species flocks with CACH and TUTI in temperate deciduous forests [11,44]. Both satellites are bark gleaners and are negatively affected by diminished access to the core species [26].

Like other core species, CACH and TUTI lead heterospecifics more often than they follow, and consistently flock with both conspecifics and heterospecifics [10]. The presence of CACH and TUTI enhances associations between WBNU and DOWO [45], in part because both satellite species copy the foraging locations of these core species [46]. Although all members forage together [47], the key aspects of foraging ecology vary among the four species. CACH, TUTI, and WBNU scatter-hoard food [48–50], but DOWO are not known to do so [51]. Scatter-hoarding involves caching food in many dispersed and cryptic locations [52], and differences in caching niches help to lower the chance of inter-species kleptoparasitism. For instance, the relatively long bill of the WBNU allows them to store seeds in places too deep for the smaller billed TUTI or CACH to reach [52]. Additionally, a dominance hierarchy exists allowing WBNU and DOWO to usurp food sources from CACH [52]. TUTI also rank higher than CACH, and are known to act aggressively towards CACH when foraging [46].

### Description of study site locations

We conducted our research in forest remnants embedded in an agriculture-dominated landscape in the glaciated region of west-central Indiana (see S1 Fig). We chose study site locations in three sections of forest corridors managed by Purdue University: Ross Biological Reserve (40°24’ N, 87°04’ W; 37.61 ha), Martell Forest (40°26’ N, 87°02’ W; 38.72 ha), and Stephens Forest (40°40’ N, 86°37’ W; 30.04 ha). Each section contained a unique subpopulation of birds. Based on the relative distances juvenile parids typically disperse (e.g. [53]), birds occupying the Ross Biological Reserve and Martell Forest were likely from the same genetic population, whereas the Stephens Forest birds were likely genetically distinct.

Disturbance rankings of Martell and Stephens Forest sites were qualitatively based on the extent to which the integrity of the natural forest structure was altered relative to the Ross Biological Reserve. The Ross reserve (hereafter ‘undisturbed’ site) is managed for biological diversity and has been left relatively untouched since its establishment in 1949 [54]. In contrast, Martell Forest (hereafter ‘mid-disturbed’ site) is managed for invasive species control and the following practices are ongoing: cut stump treatments, prescription burns, and application of basal or foliar chemical sprays. The third site, Stephens Forest (hereafter ‘most-disturbed’ site), is a managed timber harvest property that includes three different plantation stand types: three walnut stands (cumulative area of 8.30 ha), one red oak (0.53 ha), and one yellow poplar stand (1.02 ha). The plantations were created in 1972 and thinned last in 2004. Forest surrounding the plantation stands is managed (via selective harvest) to promote uneven aged growth (cumulative area of 20.19 ha).

Two harvests took place during our study. In September 2015 (first study year), a small (< 3 ha) harvest was conducted in the mid-disturbed site on select trees that died as a byproduct of herbicide application used to kill an invasive stand of burning bush (*Euonymus alatus*). In February of 2016, a salvage timber harvest was conducted on the eastern 12 ha of the most-disturbed site to remove dead and dying ash trees infested by the emerald ash borer (*Agrilus planipennis*). Neither harvest was large enough to alter forest structure or composition at a scale relevant to our study (S1 Table).

### Forest community: Vegetation and predator surveys

#### Vegetation surveys

We conducted seasonal vegetation surveys in 2016 and 2017 along randomly-placed transects to characterize the tree communities at each of the three sites. Transects were 100 m in length and were separated from one another by a minimum of 100 m; there were 13 transects at the most-disturbed site and 12 transects each at the mid-disturbed site and the undisturbed site. We determined the relative importance (relative frequency + relative density + relative dominance) of the various tree species by means of point-quarter sampling [55] at 10 randomly selected positions along each transect, with positions spaced a minimum distance of 5 m from one another. Canopy cover was estimated at every 10 m along transects using a densitometer. The understory cover was also estimated every 10 m along transects at four heights: 0–0.6 m, 0.6–1.2 m, 1.2–1.8 m, and 1.8–2.4 m, using 4 white boards, each 0.3 m wide x 0.6 m tall, stacked on top of each other. These boards were placed 15 m orthogonal to the point-quarter transect, with a measurement taken both to the right and left of the transect (i.e. 2 points for each 10 m distance). Observers estimated the relative amount of each board that was detectable from the transect line using a categorical ranking of percent detectable: 0%, 1–25%, 26–50%, 51–75%, and 76–100%. To ensure uniform data collection among observers, observers collected data only after confirmation of 90% inter observer reliability compared with the lead field technician in charge of data collection. Thus, 22 estimates of understory cover (2 observations at each board height for 11 10-m intervals) were collected for each transect. When trees were harvested within the boundaries of our sites, we resampled the vegetation along transects located nearest the harvest area and the new measures were included in subsequent analyses (see S1 Table).

Understory cover values were averaged at each of the four heights for each transect. This gave us 12 or 13 values at each understory height within a site. We then ran a factor analysis using Standardized Regression Coefficient rotated factor pattern (Proc FACTOR in SAS v9.4) to collapse data from the four heights into two factors. Factor 1 (accounting for 58% of variance) loaded most heavily on cover at the upper three heights (0.6–2.4 m). Factor 2 (accounting for 39% of the variance) loaded heavily on cover at 0–0.6 m. The factor 1 scores for each transect were used in ANOVAs (Proc MIXED in SAS v9.4) to test for seasonal and site effects. Instead of using factor 2 as a proxy for this lowest understory height, we simply used the 0–0.6 m cover data directly in our ANOVAs to test for seasonal and site effects of near-ground cover. Our conclusions are not altered if we use factor 2 instead of height from 0–0.6 m. An ANOVA was also used to test for differences in canopy cover between sites and seasons. We used least squares means (LSMEANS within Proc MIXED) to compare season and site-specific cover estimates.

A mixed effect linear model was used to determine if the mean distance from point to nearest walnut tree in each quarter was significantly less within the plantation stands than outside of the plantation stands in our most-disturbed site. This mixed model treated each transect inside and outside the plantation stands as a replicate. With the same model structure, we also tested if the walnut tree basal area differed within the plantation stands compared to the naturally-occurring walnut trees growing outside of the plantation stands. We used least squares means and standard error (LSMEANS within Proc MIXED) to compare estimates.

#### Predator surveys and predator call rates

Forest vegetation characteristics could influence avian predator species richness that, in turn, could confound our interpretation of how our study species are affected by forest structural disturbance. To assess potential differences in predator species composition across our sites, we conducted passive acoustic recording (i.e. deployed five automated recording devices (ARDs) at fixed locations within each site, see S1 Appendix for details) for a 0.5 hr around the time of dawn at least two weeks per month. Semi-automated acoustic scan sampling of known avian predators of small birds was used to detect predator calls within the acoustic files; species identity for each call was provided by author KG (see S2 Appendix for details). Of the known avian predators, Northern saw-whet owl (*Aegolius acadicus*), Cooper’s hawk (*Accipiter cooperii*), sharp-shinned hawk (*Accipiter striatus*), and Eastern screech owl (*Megascops asio*) are most likely to prey on Carolina chickadees and tufted titmice [56–58]. We also used opportunistic avian predator detections during other aspects of our field data collection and owl-banding and mist-net capture observations (personal communication with John B. Dunning).

We also considered the fact that call rates of raptor species (avian predators) determine perception of predation risk in song birds and consequently influence settlement patterns, reproductive effort, and feeding behavior [59]. We used our acoustic scan sampling results to index predator risk perception and test the possibility that any site differences in fat loads, foraging time budgets, and mixed-species flocking behavior were nonsumptive predator effects rather than energetic-related responses to habitat disturbance. We converted the acoustic scan data into a binomial “yes”/”no” variable based on the presence or absence of any raptor call during the 0.5 hr recording (irrespective of number of scan detections per audio file) and counted the number of audio files with “yes” data. We tested the binomial variable using a repeated measures Poisson regression (Proc GLIMMIX) with ARD identity as the subject variable. We tested all acoustically detected raptors separately and treated site and month as independent variables since the vocal behavior of each raptor species changes with time of year [60–62]. We also tested the cumulative call rate of all raptors in our sample. For this analysis, the 0.5 hr recording was treated as a “yes” if any call from any raptor was detected. In all models, we also corrected for the onset time of the recording relative to true dawn in the statistical model by adding time-since-sunrise as a covariate, as light influences raptor activity and calling rates as well [63,64]. Sunrise times at West Lafayette, Indiana (for the undisturbed and mid-disturbed sites) and Delphi, Indiana (for the most-disturbed site) were taken from a website (https://sunrise-sunset.org/us/lafayette-in; https://sunrise-sunset.org/us/delphi-in), and the covariate was taken as the difference between the onset of the 0.5 hr dawn recording and the sunrise time. Similarly, we corrected for the phase of the moon by treating phase as a dummy variable ranging from 0 (new moon) to 1 (full moon). The 4 phases of the moon (new, first quarter, full, third quarter) at Lafayette Indiana were taken from a website (https://www.timeanddate.com/moon/phases/usa/lafayette-in). The position of the moon between these phases was linearly interpolated. The models included moon phase, time-since-sunrise, and a month x moon phase interaction term. When the interaction was non-significant (P>0.05), it was excluded from the model structure.

### Metrics used to infer responses to habitat disturbance

#### Foraging time budget

We periodically captured and individually marked a total of 399 CACH and TUTI starting in winter 2015 through spring of 2016 and the number of marked birds ranged from 35–78 per site (see S2 Table). Birds were caught in treadle traps baited with sunflower seed four days before traps were set for capture and marking. Each bird was given a numbered, aluminum U.S. Fish and Wildlife Service band and a unique color combination of plastic leg-bands. We used strips of colored electrical tape placed over the colored band that extended about 10 mm behind the leg to facilitate identification of the birds, creating a more visible marker. No feeder stands were stocked with seeds outside of the trapping periods. The same banding procedure was followed for our satellite species, DOWO and WBNU, though they were trapped less frequently (10–12 DOWO and 37–49 WBNU banded per site compared to 47–78 CACH and 54–76 TUTI). All protocols for handling, banding, and observing animals were approved by the Purdue Animal Care and Use Committee (PACUC no. 1306000883).

Focal observations were collected during encounter surveys and flock scan samples (as described below in the Survivorship and Abundance sections, respectively). For the focal observations, actions considered as foraging behavior included pecking, food handling, bill wiping, and carrying food. We ultimately disregarded any focal samples that lasted less than four minutes and grouped focal samples according to time of year (hereafter “time frame”; time frames included November through February, representing winter when foraging energetics are most severe; March through June, representing breeding and associated offspring food provisioning; and July through October, representing favorable foraging conditions) [65,66]. For each individual, we summed the amount of time spent foraging across multiple focal samples per time frame. We also calculated the total amount of time the bird was observed in a given time frame. We divided the total amount of time each bird was observed foraging by the total amount of time the bird was observed irrespective of its behavior. We then used linear mixed models to test whether the proportion of time that we observed the birds foraging differed among sites, including ‘time frame’, ‘species’ and ‘site’ as fixed effects with ‘individual bird’ as the random intercept to account for repeated measures of the same bird (Proc MIXED in SAS v9.4). The proportions were arcsine square root transformed to fit a normal distribution. We used least squares means ± SE to evaluate differences in proportion of time spent foraging between species, time frames, and sites.

#### Home range size

We used the GPS coordinates taken each time a banded bird was positively identified during encounter surveys and flock scan samples (as described below in the Survivorship and Abundance sections, respectively) to determine if the home range of TUTI and CACH differed among our three sites. Specifically, the GPS coordinates were used to obtain a 95% fixed kernel density estimate of home range size using “KernSmooth" package in R v. 3.4.0 [67–69]; the "ks" package was used to obtain optimized KDE Plugin bandwidths. Home range size was considered separately for breeding season (February 15^th^ through August 31^st^) and nonbreeding season (September 1^st^ through February 14^th^) (see [70] for a description of seasonal patterns of Carolina chickadees in this area). Birds were excluded from home range analysis if there were five or fewer sightings in each season (54 CACH and 61 TUTI individuals were excluded from breeding season analysis, and 87 CACH and 90 TUTI individuals were excluded from nonbreeding season analysis). The home range values were averaged across years if a bird was sighted five or more times in multiple years of the same season type. If a bird was sighted more than once per day, only coordinates of the first sighting location were used in the home range analysis.

We tested whether home range size differed among sites during the breeding or nonbreeding season, accounting for potential species and sex effects. We fit two linear models using the ‘car’ package in R [71], each with the same fixed effect structure: ‘site’, ‘species’, and ‘sex’. We kept an additive model structure after confirming that model fit was not improved by a three-way interaction term or any two-way interaction terms (i.e. AIC increased with addition of interaction term). The additive models were validated through visual inspection of QQ-plots of model residuals and model assumption test results confirmed the appropriateness of a square-root transformation of home range size: Shapiro-Wilk normality test (nonbreeding: W = 0.98, P = 0.47; breeding: W = 0.99, P = 0.37); F-tests of residuals and fitted residuals that tested for homoscedasticity (nonbreeding: F_1,70_ = 0.02, P = 0.88; breeding F_1,96_ = 0.03, P = 0.87; Faraway 2005); VIF test for factor variables (GVIF < 2 in all cases); and Durwin-Watson test of non-substantive correlation among residual errors (nonbreeding: DW = 1.87, P = 0.18; breeding: DW = 1.89, P = 0.17). When appropriate, Tukey multiple comparisons of means with a 95% family-wise confidence interval was used to assess significant differences among factor levels.

#### Fat scores

Body measurements, including fat scores, were taken according to [72] and [73] when any of our four species were banded. To ensure uniform data collection among technicians, a technician only collected banding data after confirmation of 90% inter observer reliability with the lead field technician in charge of data collection. We used fat scores based on visible subcutaneous fat deposits to test if fat deposits differed across our sites. Fat scores provide a quantitative estimate of fat deposit; scores are ranked 1–8 in order of increasing fat content [73]. However, we recorded no fat scores greater than ‘4’. As the fat score is an ordinally-scaled variable, we used the polr function in the MASS package in R [74] to perform a proportional odds logistic regression model for ordinal responses in ordinal logistic regression. We treated fat score as the outcome, and site, species and season (breeding = 2/15–8/31, nonbreeding = 9/1–2/14) as additive, categorical predictors. Deviance tests confirmed that interaction terms did not significantly improve model fit (all P > 0.05). We therefore proceeded with the additive model using the R multcomp package [75] to report multiple comparisons of means using Tukey contrasts (alpha = 0.05). We also used the predict function in the MASS package to compute predicted probabilities of fat scores for CACH, TUTI, WBNU and DOWO during the nonbreeding and breeding season at each site.

#### Fledgling count

We used fledgling number to approximate reproductive success amongst sites. During the summer of 2017, encounter surveys were conducted (as described below in the Survivorship section) and incidental sightings of fledglings were also recorded. Birds were identified as fledglings if they were observed begging, emitting begging calls, or being fed by adults. Fledgling number was assessed when one or more of the parents were banded (and thus individually identifiable), or in one instance, when a pair of non-banded parents was observed simultaneously exhibiting parental behavior to the same fledgling group. Fledgling count data were not included in the analysis if there was only a single, non-banded parent present. If only one parent was observed in more than one family of the same species in a given site, we checked the GPS coordinates to make sure they were never sighted within the same territory as a putative non-mate. The same parents were all spotted with fledglings on several occasions, so it was possible for a mated pair’s fledgling count to change throughout the summer. For this reason, we recorded fledgling number as the maximum number of fledglings seen per pair.

We tested for species and site variation in fledgling number using a generalized linear model (Proc GLIMMIX in SAS v9.4). We used AIC values to find the distribution parameter that best fit the models. The tested distributions included gamma, poisson, exponential, negative binomial, normal, lognormal, geometric, and t-distribution. The t-distribution resulted in the best fit and is therefore the basis of the analysis of fledgling numbers given here. ‘Species’, ‘site’, and their two-way interaction were treated as categorical fixed effects in the model. We used least squares means ± SE to evaluate differences in fledgling number between species and sites.

#### Survivorship

Survivorship data were collected through encounter surveys, which were conducted during the breeding/spring season from February 15th through June 1st in 2016 and 2017 (see [70] for a description of seasonal patterns of Carolina chickadees in this area), as well as during the nonbreeding/winter season from September 1st through December 1st in 2015–2017. The surveys were conducted during weekdays between 0800 h and 1400 h on days without precipitation. The sites were visited on a rotating basis so that a site was never sampled on consecutive survey days. The surveys were conducted across sub-area plots that were delineated within each site based on topography, private properties lines, and trails. The order in which each plot was surveyed was randomized at the time of arrival to the site each day. Observers spent approximately 45 minutes within each plot looking and listening for the birds. Each time a bird was encountered and positively identified, its location was recorded using a Garmin Etrex 20 GPS device. For each survey interval, encounter data were entered as “1” if a banded bird was sighted (including the date the bird was banded), or “0” if a banded bird was not sighted.

We excluded juvenile birds from the dataset due to the propensity of birds from both species to disperse from the natal environment before adulthood [65,76]. We also excluded birds that were never sighted again after banding to eliminate transients from our dataset. If a bird was banded prior to adulthood, encounter data were not recorded until the bird was resighted as an adult (any time after its first winter; S2 Table). If a bird was banded in between survey intervals, encounter data were entered for the previous survey.

We used the R package RMark to model species-specific apparent survival (Φ) and detection probabilities (p) [77]. We followed an information theoretic approach and multi-model inference to account for model selection uncertainty and to obtain robust parameter estimates [78]. We constructed a candidate model set of 21 Cormack-Jolly-Seber models (S3 Table). Survival and detection probabilities varied by group (site), season (winter, spring), and survey occasion (‘time’). Candidate models with interaction terms and constant detection probabilities were also included in the candidate set. We did not include effects of sex to avoid overparameterization, but also because previous studies show sex does not significantly influence survival in parids [79–81].

We calculated the variance inflation factor (ĉ), or reduced chi-square, for a fully study-site and time-dependent global model using the median c-hat approach in Program MARK to estimate over-dispersion [82,83]. In the case of over-dispersion (ĉ > 1), AICc values were adjusted through quasi-likelihood (QAICc), otherwise candidate models were ranked using the Akaike’s Information Criterion for small datasets (AICc). Apparent survival estimates and detection probability estimates were model-averaged across candidate models with a cumulative ≤ 0.95 weight and are reported with unconditional standard errors (estimate ± SE).

#### Abundance

We used flock scan samples and encounter survey data collected between March 1st through April 19th, 2016 as mark-resight data in Program Mark to obtain population abundance estimates for CACH and TUTI [83]; no additional birds were banded during this time frame. Scan sampling followed the same procedure used for encounter surveys, and approximately 50 hours were spent collecting scan sample data at each site. Each time a flock was sighted, banded birds were identified and the number of nonbanded CACH and TUTI was recorded. We did not assume demographic closure, as examination of the number of resightings per banded individual revealed not all birds were resighted multiple times between March 1^st^ and April 19^th^, 2016. However, the low proportion (9/73) of birds seen only once indicates that the effect from dispersal and death is negligible. We also did not assume that the study population was geographically closed, as our sites represented a portion of a linear continuous forest patch corridor. We sampled with replacement, as it was possible for the same birds to be sighted more than once in a day. We also included individuals in the analysis as “+0” if they were seen incidentally outside of scan sampling to set a more accurate minimum population size of birds known to persist in the study area during that time [84]. We therefore used a Poisson mark-resight model, as it accounts for the fact that 1) CACH and TUTI are highly mobile, and 2) the exact number of marked individuals in the population at the time of resighting surveys was unknown. The Poisson mark-resight approach also allows for the inclusion of resight data from partial color band readings, where an individual is identified as marked, but not to individual identity.

We constructed a set of candidate models for each species and considered the influence of site (group effect) on male population abundance (S4 Table). Candidate models with constant and group dependent parameters were included to account for potential differences in individual resighting heterogeneity, survival rates, resighting probability, and number of unmarked individuals among sites. An information theoretic approach and multi-model inference was used to account for model selection uncertainty and to obtain robust parameter estimates. Candidate models were ranked using the Akaike’s Information Criterion (AIC) [78]. Derived parameter estimates were model-averaged across all candidate models and results were interpreted based on the weighted average estimate, unconditional standard errors (SEs), and 95% confidence intervals. We accounted for the possibility of movement across site boundaries by interpreting the abundance estimate as that of the ‘super population’, or the number of individuals associated with the study area during the seven-week time frame. We also consider the abundance estimates per the hectare size of each site (see “Description of Study Site Locations” section above).

#### Flock size and species composition

Flock size (number of members in a flock) and species composition were recorded during flock scan samples at each of the three sites from October 2015 through June 2017 (for a description of encounter surveys and flock scan samples, see respective Survivorship and Abundance sections above). Flocks were defined as the spatial association of at least two birds who were at most 10 m from the next closest bird and traveling in the same direction. Waiting 5 min after locating a flock, the technician would identify each bird in the flock by species and band color if the bird was banded. Flock size and composition were estimated every 5 min during a focal animal sample on a banded bird. Flock numbers collected over the course of a single focal animal sample were averaged so that a single flock structure was used for each focal animal sample.

The data were analyzed using repeated measures Poisson regressions (Proc GLIMMIX in SAS v9.4). For the flock size analysis, we built a model that included ‘site’, ‘time’, and their two-way interaction in the fixed effect structure. ‘Time’ was categorized according to time of year, including ‘non-breeding’ (September through February), and ‘breeding’ (March through August). The year was broken up into two periods to ensure a reasonable estimate of space use for statistical replicates (see below). For the flock composition and association analysis, we used the regression coefficient (ß) to test whether the ‘number of satellite species’, and ‘number of TUTI’ explained the ‘number of CACH’ in a flock. Likewise, we used the regression coefficients (ß) to test whether the ‘number of CACH’, and ‘number of satellite species’ explained the ‘number of TUTI’ in a flock. Finally, we used the regression coefficients (ß) to test whether the ‘number of CACH’, and ‘number of TUTI’ explained the ‘number of satellite species’ in a flock. As with the flock size analysis, we included ‘time’ and ‘site’. We tested all possible interactions between the four fixed effects; non-significant interaction terms were removed in order of decreased F value until all interaction terms left in the model were significant (P<0.05). We tested for the effect of ‘time’ in case disturbance resulted in extended mixed-species flock formation during the breeding season.

Replication of flock-level measures for each site was determined quantitatively based on overlap of spatial distribution of banded birds. We used a spatial cluster analysis (Proc FASTCLUS in SAS 9.4) of the location of banded birds observed during the flock scan samples to identify three sections in each site with minimal interchange of birds (miss-classification < 9%), providing N = 3 independent measures of flock-level characteristics per site. As discussed above, each year (2015/16 and 2016/17) was divided into two time intervals (breeding and non-breeding); dimensions of the three site-specific sections were calculated separately for each year and time interval. We did not break each year into more intervals to ensure that we had a sufficient sample size to identify areas with minimal interchange of birds. The spatial dimensions of each cluster were estimated using discriminant function analysis (Proc DISCRIM in SAS 9.4).

## Results

### Forest vegetation

Importance values were highest for maples and walnuts at the most-disturbed site, whereas oak and maple, and poplar and maple had highest importance values at the mid-disturbed and the undisturbed site, respectively (see S5 Table). Walnut tree basal area at the most-disturbed site was significantly smaller within the plantation stands (17.32 ± 2.04 cm^2^) than outside of the plantation stands (32.60 ± 2.23 cm^2^; F_1,5.42_ = 25.47, P = 0.003). In addition, the mean distance from point to nearest walnut tree in each quarter at the most-disturbed site was significantly less within the plantation stands (3.52 ± 0.31 m) than outside of the plantation stands (5.56 ± 0.54 m; F_1,10.7_ = 10.63, P = 0.001), indicating that the density of smaller walnut trees is higher within the plantation stands.

Canopy cover did not significantly differ among sites (F_2,115_ = 2.07, P = 0.13), but did change with season (F_3,115_ = 2159.20, P = <0.0001). Canopy cover was thinner in the spring (13.88 ± 0.83%) and winter (16.65 ± 0.84%) than in summer and fall (82.71 ± 0.84% and 82.67 ± 0.84%, respectively). There was a seasonal effect but no site effect on the understory cover factor 1 scores (i.e., understory cover between 0.6 to 2.4 m height; season: F_3,109_ = 17.18, P = 0.0001; site: F_2,109_ = 1.73, P = 0.18). In contrast, both seasonal and site effects were significant with understory cover from 0–0.6 m (F_3,109_ = 66.79, P = < 0.0001; F_2,109_ = 8.79, P = 0.0003, respectively); understory cover (arcsin square root transformed) from 0–0.6 m was thinner in the spring (1.14 ± 0.07) and winter (1.35 ± 0.07) compared to the summer and fall seasons (2.68 ± 0.07 and 2.53 ± 0.07, respectively). Finally, the understory cover at 0–0.6 m was thickest at the most-disturbed site (2.17 ± 0.07) compared to the undisturbed or mid-disturbed sites (1.69 ± 0.07 and 1.91 ± 0.07, respectively).

### Predator survey and call rates

Results from acoustic surveys, incidental field sighting reports, and owl banding and mist-net capture data strongly suggest that raptor species richness varies minimally amongst our sites. Specifically, presence within the boundaries of each of our site locations was confirmed for the following resident raptor species: barred owls (*Strix varia*), eastern screech owl (*Megascops asio*), great-horned owl (*Bubo virginianus*), red-shouldered hawk (*Buteo jamaicensis*), Cooper’s hawk (*Accipiter cooperii*), and sharp-shinned hawk (*Accipiter striatus*). Presence of the Northern saw-whet owl (*Aegolius acadicus*), a migratory species known to occupy forest habitat in winter throughout the state of Indiana [85,86], was confirmed at both the mid-disturbed and undisturbed sites.

**Fig 1:**
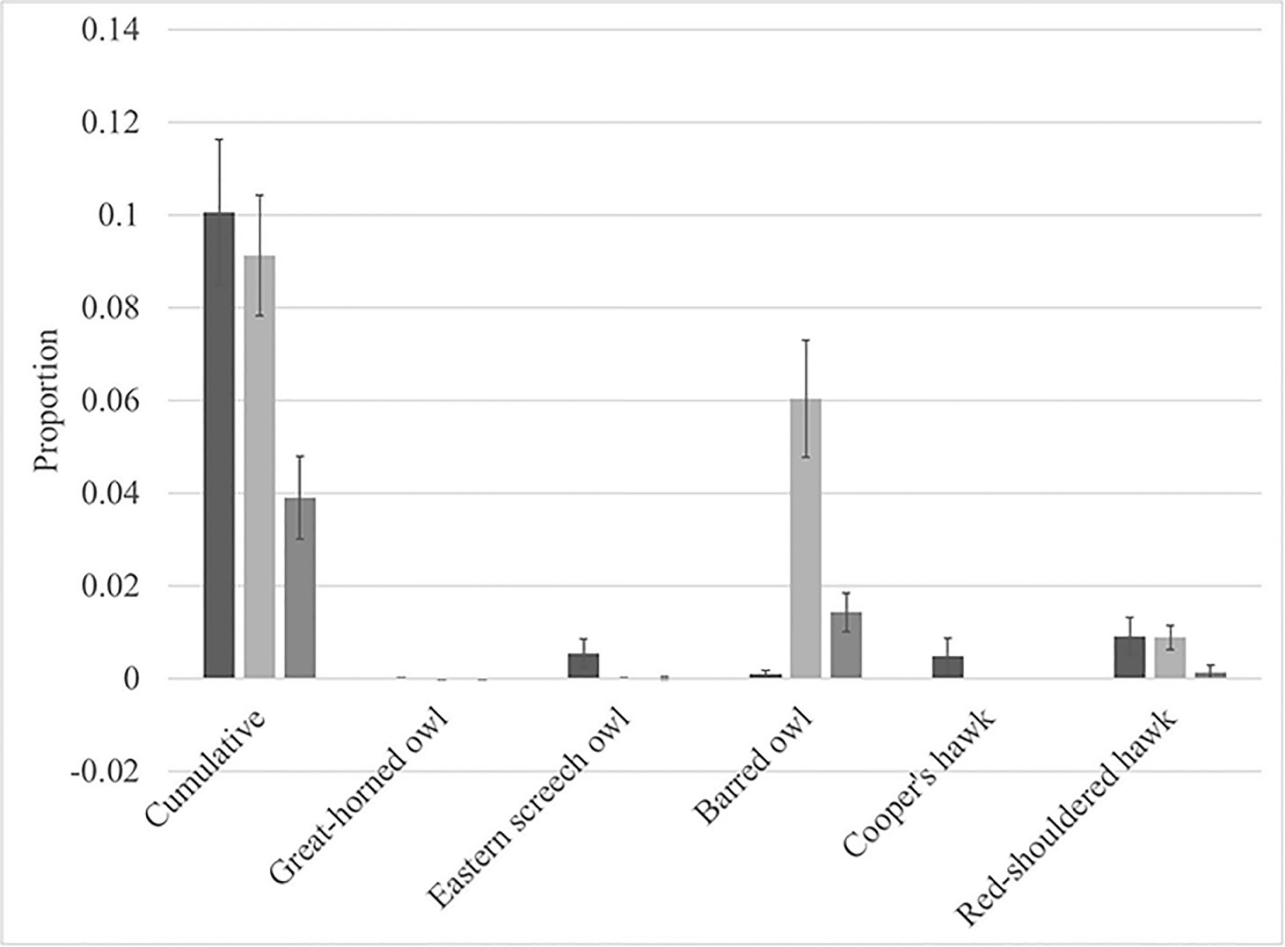
Proportion (Mean ± SE) of samples that contained at least one raptor call. Sample represents a 30 minute acoustic recording. Data for individual owl species are least means squares (see text); means/standard error otherwise derived from raw data. Undisturbed site data is represented by the left bar (dark grey), mid-disturbed site data is represented by the middle bar (light grey), and most-disturbed site data are represented by the right bar (second lightest shade of grey).

The cumulative raptor species call rate (F_2,12_ = 8.35, P = 0.0053) and individual owl species call rates varied significantly between sites (Fig 1, Table 1). There was also a significant interaction between moon phase and month in the cumulative raptor call rate model (F_11,2027_ = 2.00, P = 0.0249), though neither of the main effects were significant (moon: F_1,2027_ = 1.97, P = 0.1608; month: F_11,148_ = 1.77, P = 0.0642). Raptor call rates also did not vary with time since sunrise (F_1,2027_ = 2.24, P = 0.1346). The GLIMMIX models for Cooper’s hawks and red shouldered hawks failed to converge. Nonetheless, the raw call rates for these hawks followed the owl pattern: red shouldered hawk calls were lowest at the most-disturbed site and no Cooper’s hawk calls were recorded at the most-disturbed site (Fig 1).

**Table 1:** Results for Type III Tests of fixed effects for raptor call rate models. Significant P values are bolded. ‘NS’: non-significant interaction that was excluded from the fixed effect structure (see Methods).

**Great horned owl**				
**Site**	**Month**	**Time since sunrise**	**Moon**	**Moon x month**
F_2,12_ = 25.37; **P < 0.0001**	F_11,148_ = 2.44; **P = 0.0080**	F_1,2027_ = 1.81; P = 0.1786	F_1,2027_ = 0.00; P = 0.9998	F_11,2027_ = 4.32; **P < 0.0001**
**Screech owl**				
**Site**	**Month**	**Time since sunrise**	**Moon**	**Moon x month**
F_2,12_ = 18.21; **P = 0.0002**	F_11,148_ = 2.92; **P = 0.0016**	F_1,2027_ = 6.99; **P = 0.0082**	F_1,2027_ = 0.20; P = 0.6584	F_11,2027_ = 4.10; **P < 0.0001**
**Barred owl**				
**Site**	**Month**	**Time since sunrise**	**Moon**	**Moon x month**
F_2,12_ = 43.21; **P < 0.0001**	F_11,148_ = 3.77; **P < 0.0001**	F_1,2026_ = 7.32; **P = 0.0069**	F_1,2026_ = 1.26; P = 0.2614	F_11,2026_ = 3.63; **P < 0.0001**

### Foraging time budget

Proportion of time spent foraging changed with season (F_2,89.2_ = 19.77, P < 0.001), with the greatest proportion occurring during the winter months (November through February; 0.62 ± 0.03). Proportion of time foraging did not significantly differ between the other two time frames (β_diff_ ± SE = -0.07 ± 0.05). In addition, the proportion of time that birds spent foraging differed among sites (F_2,63.9_ = 10.75, P < 0.0001). Specifically, birds at the most-disturbed site spent significantly more time foraging (0.58 ± 0.04) relative to the mid-disturbed site (0.38 ± 0.03; t_62.6_ = -4.63, P<0.0001) and undisturbed site (0.48 ± 0.03; t_66.5_ = -2.30, P = 0.025). The proportion of time spent foraging was lower at the mid-disturbed site compared to the undisturbed site as well (β_diff_ ± SE = -0.10 ± 0.04; t_62.9_ = -2.4, P = 0.018). Species also differed in the proportion of time spent foraging (F_1,74.8_ = 5.70, P = 0.02), with CACH spending a higher proportion of time compared to TUTI (0.53 ± 0.02; 0.44 ± 0.03, respectively).

### Home range size

Ninety-eight birds (36 CACH + 27 TUTI males and 17 CACH + 18 TUTI females) were opportunistically sighted during the breeding seasons. In addition, 72 birds (28 CACH + 17 TUTI males and 16 CACH + 11 TUTI females), were opportunistically sighted during the nonbreeding seasons. Both the nonbreeding and breeding additive model structures were significant (F_4,67_ = 4.89, P = 0.002, adjusted R^2^ = 0.18; F_4,93_ = 3.44, P = 0.011, adjusted R^2^ = 0.09, respectively).

**Fig 2:**
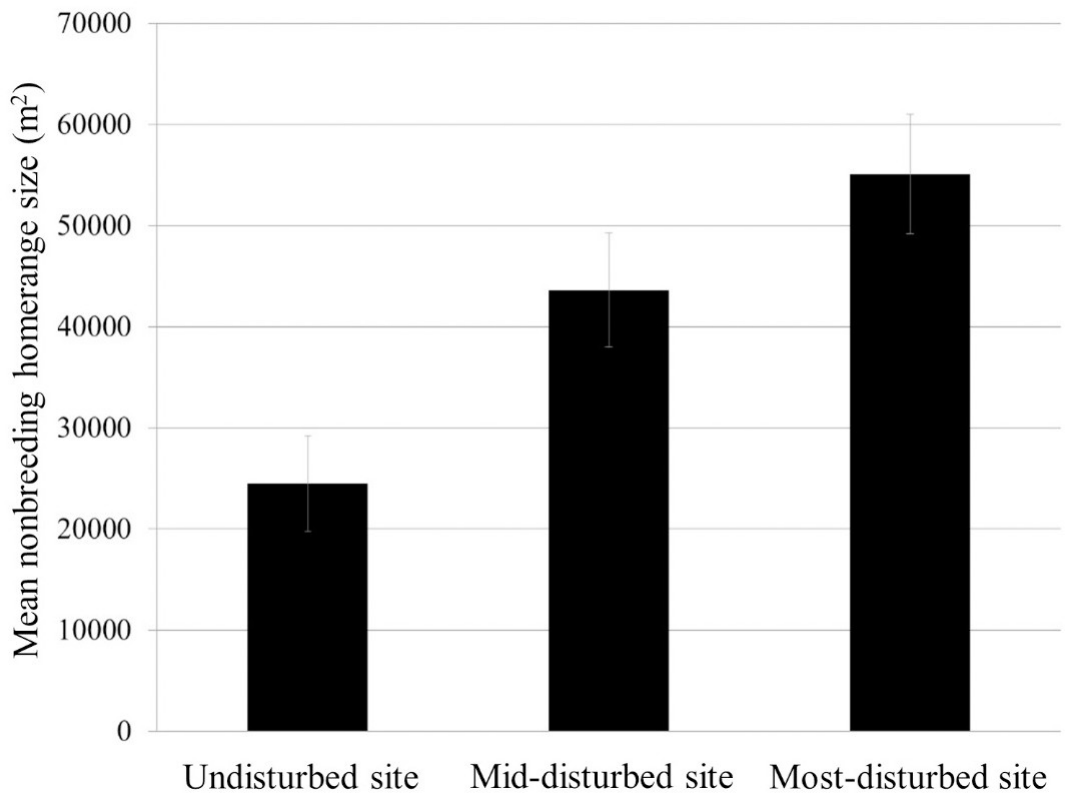
Mean (± SE) home range size per site. Home range size was significantly smaller at the undisturbed site compared to the most-disturbed site during the nonbreeding season.

Sex did not affect home range size during the nonbreeding season (F_1,67_ = 0.96, P = 0.33). There also was no species effect on home range size during the nonbreeding season (F_1,67_ = 3.22, P = 0.08). However, home range size did differ among sites (F_2,67_ = 7.69, P = 0.001). In particular, home range size was significantly larger at the most-disturbed site relative to the undisturbed site (pairwise Tukey post-hoc CI: 31.10–128.93). Home range did not differ significantly between the mid-disturbed site and the undisturbed site, or between the mid-disturbed site and most-disturbed site (see Fig 2 for site-specific mean and SE). In contrast, we detected a sex effect on home range size during the breeding season (F_1,93_ = 8.67, P = 0.004). However, there was no species effect (F_1,93_ = 0.30, P = 0.584). Finally, breeding season home range size did not differ among sites (F_2,93_ = 2.40, P = 0.097).

### Fat scores

**Fig 3:**
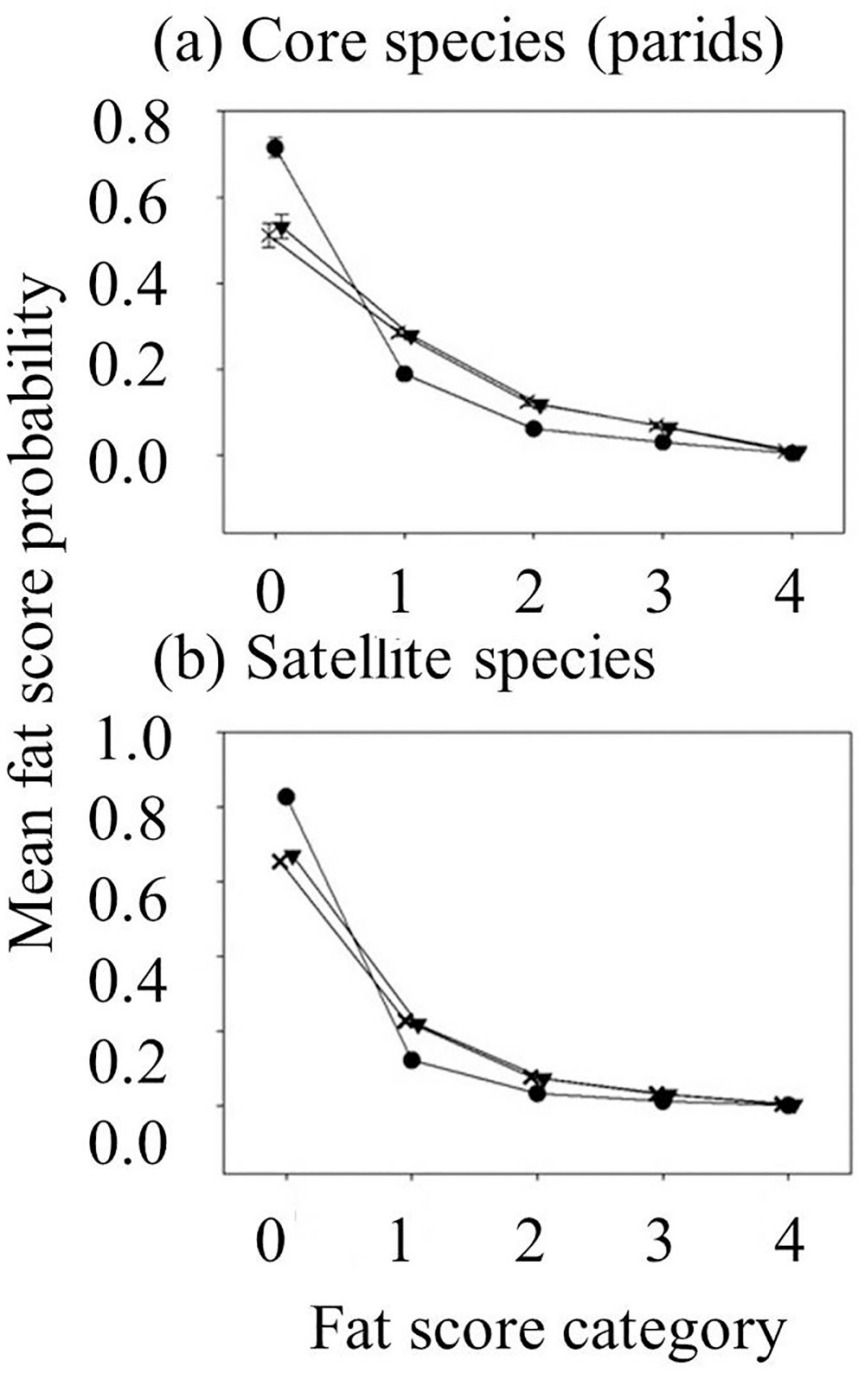
Probability distribution of fat scores for (a) parid species (CACH and TUTI) and for (b) satellite species (WBNU and DOWO). Means + SD are given. Fat score probabilities are averaged across season and species. Both groups have a higher probability of a fat score of ‘0’ (no visible fat) and a lower probability of fat accumulation (scores 1–4) in the most-disturbed site (closed circles) compared to the mid-disturbed site (closed triangles) or the undisturbed site (X’s). The values within each fat score are offset to facilitate comparisons between sites.

The fat scores measured at the undisturbed site were significantly higher than those at the most-disturbed site (β_diff_ ± SE = 0.94 ± 0.25, z = 3.81, P = 0.0004). The fat scores were also higher at the mid-disturbed site relative to the most-disturbed site (β_diff_ ± SE = 0.87 ± 0.25, z = 3.52, P = 0.0013). In contrast, fat scores did not vary significantly between the mid-disturbed site and the undisturbed site (multiple comparisons of means, Tukey contrasts, P = 0.931). WBNU fat scores were significantly less than those of TUTI and CACH (β_diff_ ± SE = -0.86 ± 0.26, z = -3.27, p = 0.005; β_diff_ ± SE = -0.87 ± 0.27, z = -3.29, P = 0.005, respectively). Fat scores otherwise did not vary significantly among species (multiple comparisons of means, Tukey contrasts, P = 0.999 for CACH—ETTI; P = 0.603 for DOWO—ETTI; P = 0.588 for DOWO—CACH; P = 0.770 for WBNU—DOWO). Fat scores did not significantly differ between seasons (β_diff_ ± SE = -0.32 ± 0.22, z = -1.48, P = 0.14). The probability of a fat score of ‘0’ was greatest at the most-disturbed site, and the probability of all other scores of fat accumulation was relatively higher at the undisturbed site and the mid-disturbed site compared to the undisturbed site (Fig 3).

### Fledgling counts

Similar numbers of families were observed at the undisturbed site and the mid-disturbed site, including 7 TUTI families and 4–5 CACH families. Five TUTI families and only a single CACH family were observed at the most-disturbed site.

**Fig 4:**
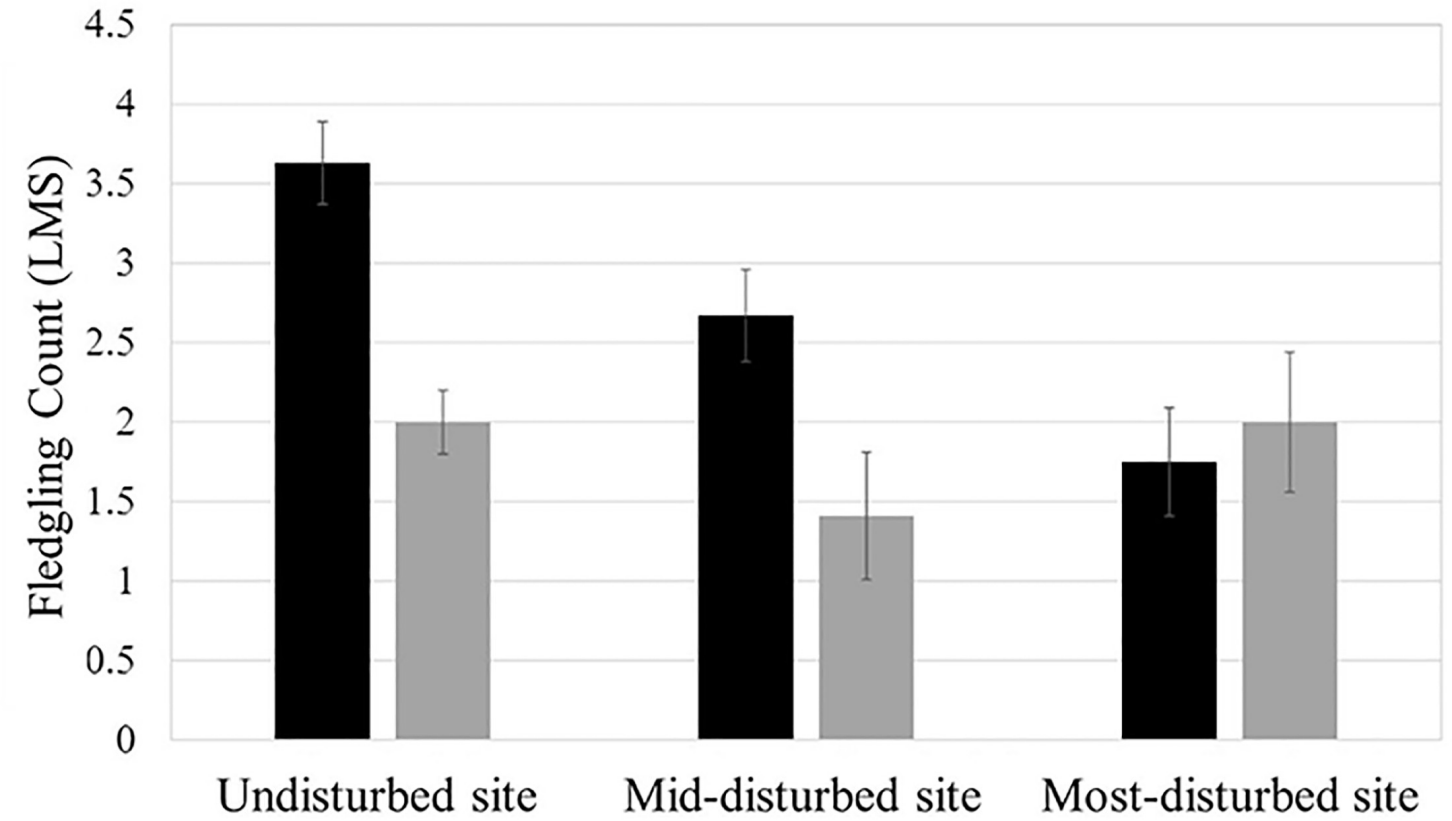
Least mean squares (LMS±SE) fledgling count estimates. For each site, TUTI data are represented by the left bar (black), while CACH data are represented by the right bar (grey).

The relationship between site and fledgling count differed between species, as indicated by a significant species and site interaction (F_2,23_ = 4.26, P = 0.03). For TUTI, fledgling count significantly differed among sites (Fig 4). Specifically, TUTI fledgling count was lower in the mid-disturbed site and the most-disturbed site relative to the undisturbed site (mid-disturbed site vs. undisturbed site: β_diff_ ± SE = −0.96 ± 0.38; undisturbed site vs. most-disturbed site: β_diff_ ± SE = 1.88 ± 0.43). In contrast, CACH fledgling count did not differ significantly among sites, which is in large part related to the lack of data from the most-disturbed site.

### Survivorship

Of the 199 TUTI banded, only 41% were encountered again after banding, compared to 54% of the 200 CACH banded (S2 Table). Initial attempts to include CACH from the undisturbed site in the survivorship analysis failed, as the parameters could not be estimated due to issues regarding the characteristics of the encounter data (e.g. small sample size). For this reason, we excluded CACH from the undisturbed site from the analysis, and thus only report and compare apparent survival and detection probabilities for CACH from the mid-disturbed and most-disturbed sites.

**Fig 5:**
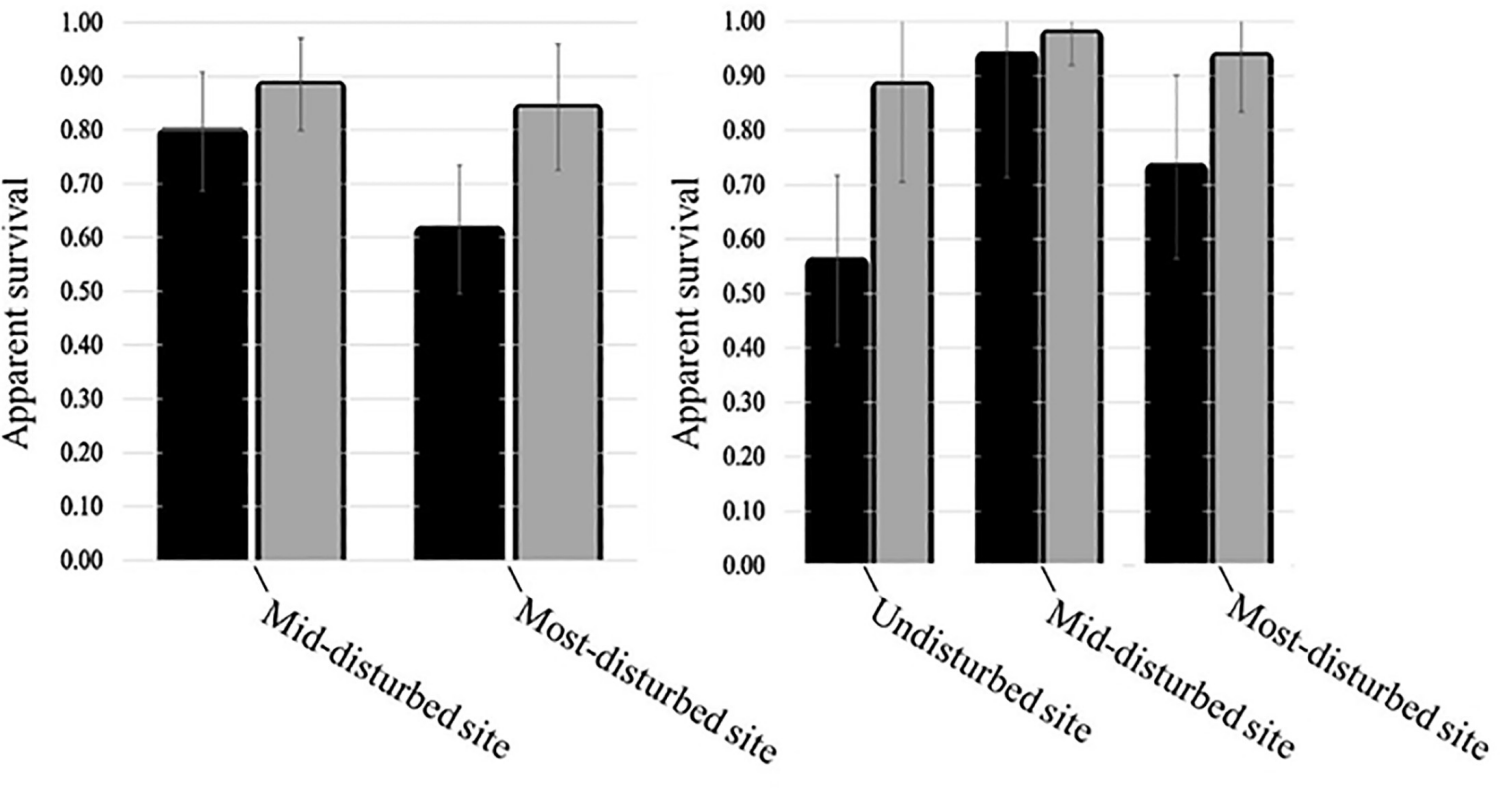
Site-specific apparent survival estimates (± unconditional SE). (left) CACH survival estimates, and (right) TUTI survival estimates. The darker shaded bar represents non-breeding season and the lighter color bar represents breeding season in each graph. Note: no survival estimates are available for CACH at the undisturbed site.

Seven candidate models were averaged for CACH and five model were averaged for TUTI (cumulative weight ≤ 0.95; S6 Table). The apparent survival estimates changed with site and season for both species, whereas detection probability estimates changed with sampling period and site. We found that survival was lower for both TUTI and CACH at the most-disturbed site compared to the mid-disturbed site (Fig 5). However, TUTI survival estimates were lowest at the undisturbed site compared to the other two sites. For both species, survival was lower during the non-breeding interval relative to the breeding interval, irrespective of site, and apparent survival was higher at the mid-disturbed site compared to the most-disturbed site regardless of season. The difference in survivorship between sites was greater in the non-breeding season than it was in the breeding season (Fig 5).

Detection probabilities for both species tended to be highest in spring (February 15th to June 1st; S7 Table). For CACH, detection probability was higher at the most-disturbed site compared to the mid-disturbed site. Except for the fourth sampling period (non-breeding, 2016/17), CACH detection probability at the most-disturbed site was also higher than detection probabilities for TUTI regardless of site. In contrast, detection probabilities were similar for TUTI at the mid-disturbed site and the most-disturbed site, but higher at the undisturbed site.

### Abundance

The abundance estimates (i.e. number of individuals estimated to be associated with the study area during our seven-week time frame) were lowest at the most-disturbed site for both species (see Table 2a and 2b). However, the relative abundance of CACH at the mid-disturbed site was very close to the abundance at the most-disturbed site (0.49/ha vs. 0.48/ha, respectively). Relative abundance of CACH was highest at the undisturbed site (0.56/ha). In contrast, the abundance of TUTI was highest at the mid-disturbed site (0.40/ha, 0.65/ha, 0.56/ha: most-disturbed, mid-disturbed, and undisturbed site listed respectively).

**Table 2:** Sample summary and Poisson mark-resight model-averaged derived estimates for CACH and TUTI abundance. The total number of marked individuals resighted at least once and known to be in the population (n*_j_), the total number of unmarked individual sightings (*T*_uj_), and the total number of times an individual was sighted and identified as marked, but not identified to individual identity (∊_j_). Estimate of ‘super population’ size ($\hat{N}$), overall mean resighting rate estimate (Lambda-hat), overall probability of being captured 1 or more times ($\hat{\text{p}}$).

Species	Site	n*_j_	*T*_*ni*_	∊_j_	$\hat{N}\;\pm \;\text{SE}\left(95\%\;\text{CI}\right)$	Lambda-hat ± SE (95% CI)	$\hat{\text{p}}\;\pm \;\text{SE}\left(95\%\;\;\text{CI}\right)$
CACH	Undisturbed	17	28	0	20.80 ± 2.11 (16.67–24.93)	3.93 ± 0.78 (2.40–5.46)	0.91 ± 0.04 (0.78–0.96)
	Mid-disturbed	13	18	5	19.25 ± 1.79 (15.75–22.75)	3.88 ± 0.69 (2.54–5.22)	0.92 ± 0.04 (0.80–0.97)
	Most-disturbed	13	13	1	14.41 ± 1.72 (11.04–17.78)	3.62 ± 0.63 (2.38–4.87)	0.92 ± 0.04 (0.78–0.97)
TUTI	Undisturbed	12	9	0	20.72 ± 3.66 (13.55–27.88)	1.43 ± 0.37 (0.71–2.16)	0.73 ± 0.09 (0.52–0.87)
	Mid-disturbed	13	15	0	24.75 ± 4.48 (15.96–33.53)	1.87 ± 0.60 (0.75–2.99)	0.70 ± 0.10 (0.49–0.85)
	Most-disturbed	5	5	0	11.51 ± 2.83 (5.96–17.06)	1.66 ± 0.50 (0.68–2.65)	0.74 ± 0.10 (0.49–0.89)

### Flock size and species composition

We followed flocks on 658 occasions over two years and across the three sites. CACH, TUTI, WBNU, and DOWO were observed flocking together in each site. There was no significant interaction between site and season for total flock size (F_2,14_ = 2.70, P = 0.10), and total flock size did not change across the disturbance gradient (F_2,27_ = 0.28, P = 0.76). However, flock size did vary across time intervals (F_1,14_ = 45.8, P < 0.001), such that it was smaller in the breeding time interval (3.02 ± 0.11) compared to the non-breeding interval (4.08 ± 0.13).

**Fig 6:**
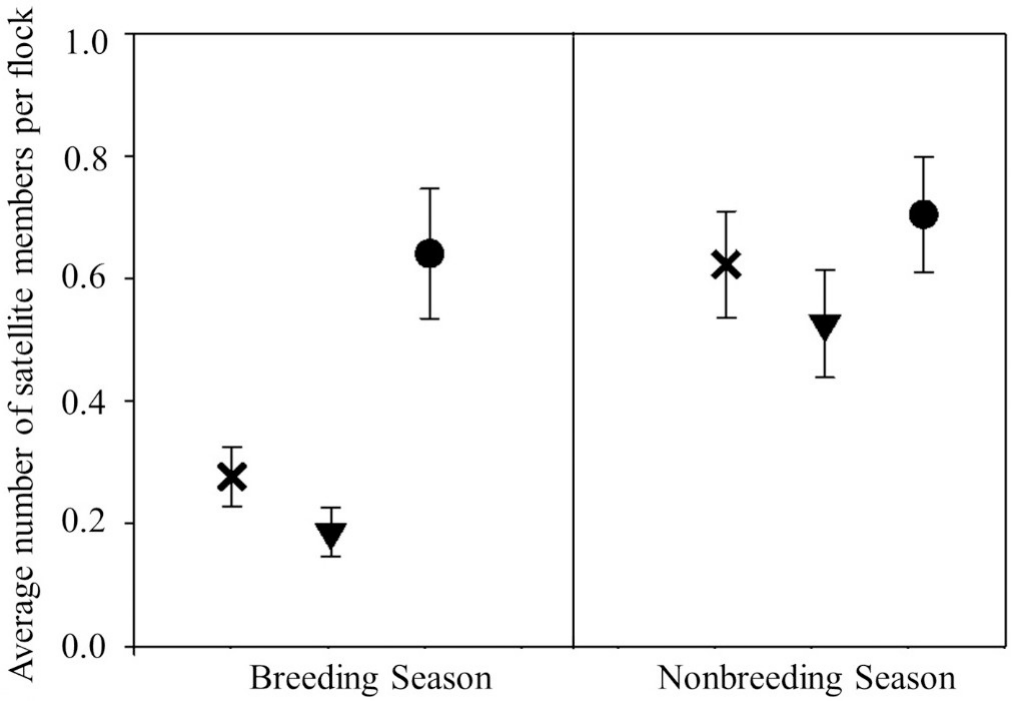
Mean ± SE derived from a Poisson regression of number of satellite members per flock at each site during breeding and nonbreeding seasons. Average number for the most-disturbed site is represented by the circle, while the triangle and ‘x’ represents the average number for the mid-disturbed and undisturbed sites, respectively. Note: numbers less than 1 suggest that not all flocks had satellites species.

Additionally, there was a significant interaction between season and site for the number of satellite members per flock (See Table 3 for results of type III tests of fixed effects). This interaction resulted from a relatively large number of satellites in flocks during the breeding interval at the most-disturbed site (Fig 6). However, the average number of satellite members was not significantly different between any of the three sites during the non-breeding interval (all P > 0.23; Fig 6).

**Table 3:** Results for Type III Tests of fixed effects for flock composition/association models. Significant P values are bolded. ‘NS’: non-significant interaction that was excluded from the fixed effect structure (see Methods). ‘Time interval’: breeding vs. non-breeding time intervals (see Methods).

**Number of satellite species in a flock**							
**Site**	**Time interval**	**Species**		**Site x time interval**	**Site x species**		**CACH x TUTI**
		**CACH**	**TUTI**		**CACH**	**TUTI**	
F_2,27_ = 10.86; **P = 0.0003**	F_1,14_ = 17.45; **P = 0.0010**	F_1,621_ = 0.09; P = 0.77	F_1,621_ = 1.13; P = 0.29	F_2,6_ = 4.89; **P = 0.025**	NS	NS	F_1,621_ = 6.48; **P = 0.011**
**Number of CACH in a flock**							
**Site**	**Time interval**	**Species**		**Site x time interval**	**Site x species**		**Satellite x TUTI**
		**Satellite**	**TUTI**		**Satellite**	**TUTI**	
F_2,6_ = 0.15; P = 0.86	F_1,8_ = 28.0; **P = 0.0007**	F_1,644_ = 0.79; P = 0.38	F_1,644_ = 59.6; **P < 0.0001**	NS	NS	NS	F_1,644_ = 7.13; **P = 0.0078**
**Number of TUTI in a flock**							
**Site**	**Time interval**	**Species**		**Site x time interval**	**Site x species**		**Satellite x CACH**
		**Satellite**	**CACH**		**Satellite**	**CACH**	
F_2,6_ = 1.99; P = 0.22	F_1,8_ = 9.17; **P = 0.016**	F_1,644_ = 2.46; P = 0.12	F_1,644_ = 108.6; **P < 0.0001**	NS	NS	NS	F_1,644_ = 11.2; **P = 0.0009**

In comparison, the number of TUTI in a flock was consistent amongst sites and the site effect did not change significantly across time intervals (i.e. no site × time interval interaction; Table 3). However, there was a significant seasonal effect: the number of TUTI per flock was highest during the non-breeding interval compared to the breeding interval (1.37 ± 0.12 vs. 0.81 ± 0.08, respectively). Similarly, the number of CACH per flock was consistent across sites with no significant site×time-interval interaction (Table 3), and the number of CACH per flock was higher in the non-breeding interval than in the breeding interval (2.08 ± 0.09 vs.1.50 ± 0.08, respectively).

There was no main effect on the number of satellites per flock of either the number of CACH or the number TUTI in the flock (Table 3). However, there was a significant effect on satellite number of the joint number of CACH and TUTI (ẞ ± SE = 0.12 ± 0.05), suggesting that flocks composed of larger numbers of both core species were attractive to satellites. The attraction of core species to satellite species did not differ between sites (F_2,620_ = 0.74, P = 0.48). The number of CACH in the flock was negatively affected by the number of TUTI per flock (ß ± SE = -0.27 ± 0.03), although a significant positive interaction term between number of TUTI and number of satellites (ß ± SE = 0.096 ± 0.036) suggests that the avoidance of TUTI by CACH is moderated with an increasing numbers of satellite individuals. The effect of TUTI on CACH did not differ between sites (F_1,622_ = 0.3, P = 0.77), nor did the interaction effect of TUTI + satellites differ between sites (F_2,620_ = 1.2, P = 0.31). The inverse relationship held true as well: flocks with relatively high numbers of CACH had fewer TUTI members (ß ± SE = -041 ± 0.04), and this effect was moderated with increasing numbers of satellites (ß ± SE = 0.14 ± 0.04). There was no significant site effect on either the CACH effect (F_1,622_ = 0.8, P = 0.46) or the CACH + satellite effect (F_2,620_ = 0.3, P = 0.71). The lack of an interaction effect between core or satellite species and site suggests the aversion between core species, and attraction of satellite species to core species, is similar across the disturbance gradient.

## Discussion

Collectively, our data suggest that core species in the most-disturbed site experienced energetic deficits; they spent more time foraging, had larger home ranges, and were less likely to have accumulated fat than parids in the less disturbed sites we examined. Satellite species were also less likely to accumulate fat in the most-disturbed habitat. Although we did not directly measure food availability, we can infer from these results that the foraging value of forest habitat declines across our disturbance gradient [87]. Moreover, it is unlikely that elevated predation risk and/or perception of predation risk at the most-disturbed site accounts for the patterns we observed, given that cumulative raptor call rates were significantly lower at this site compared to our other two sites and the call rates of individual raptor species were not higher at the most-disturbed site.

**Fig 7:**
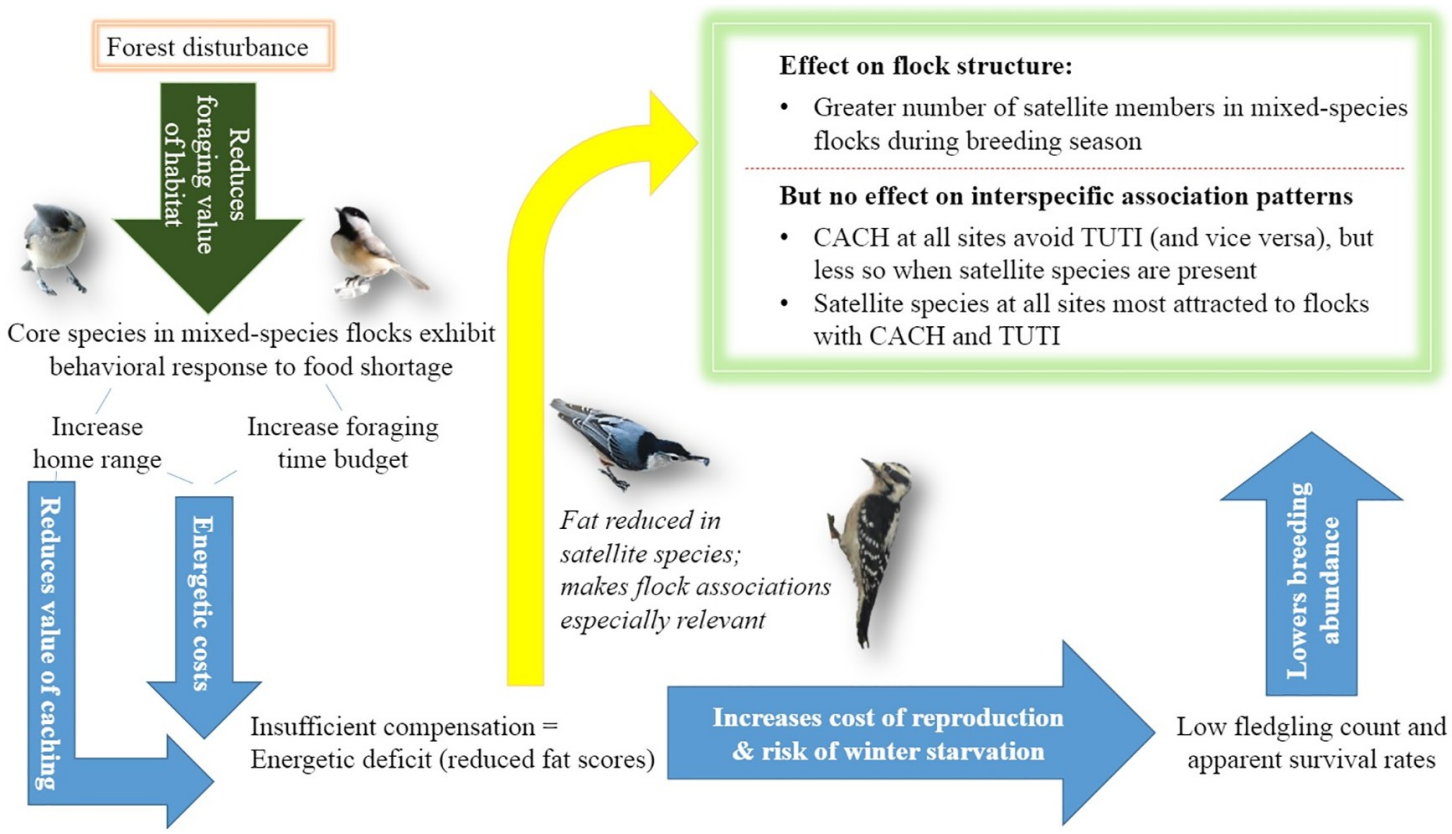
Flow diagram depicting a mechanistic chain leading from degraded forest structure to altered flock structure in the most-disturbed site. Core species respond to poor-quality foraging habitat by increasing home range and foraging time budget, which are energetically costly behaviors that can reduce the value of caching. The resultant energetic deficit in turn can increase the cost of reproduction and risk of winter starvation, potentially influencing abundance and satellite species flocking associations. Indeed, the number of satellite members in mixed-species flocks was unexpectedly high during the breeding season. Photo credit: Katherine E. Gentry.

Significant energetic constraints from a shortage of resources in the most-disturbed site likely explain the lower fledgling production and survivorship of the core species. The unexpected high number of satellite members flocking with core members during the breeding season suggests that the value of mixed-species flock associations is increased in poor-quality foraging habitat beyond the time that mixed species flocks typically form. We discuss the implications of our findings in greater detail below (also see Fig 7).

### Poor-quality foraging habitat and its implications for core species

The core species increased investment of time spent foraging, reflecting the reduced foraging value of the forest habitat in the most-disturbed site. Numerous studies indicate that an increase in time spent foraging is an index of poor foraging conditions [88–90] (also see [91]). The relationship is explained by the fact that food shortages require greater foraging effort to achieve the same energetic income (e.g. [92]). The daily increase in foraging time in turn limits the time and energy available for other critical requirements, including social interactions, predator detection, territorial defense, preening and resting [93].

Patterns in space use tend to co-vary with foraging habitat quality [94], especially when energetic costs related to increased foraging behavior reduce the economic defensibility of space (e.g. [95]). For instance, evenly distributed and predictable food resources favor small home ranges and territoriality, whereas low quality foraging habitat favors an increase in undefended home range size that allows for an expanded range of resources available to an individual [32,93]. Indeed, we found that home range sizes were largest at the most-disturbed site, which further supports the conclusion that foraging habitat value is relatively degraded at the most-disturbed site. The increase in space use can result in several additional consequences, including the number of foraging-related costs such as locomotory costs, and costs associated with assessing, learning and relocating food supplies within the home range [32].

### Low fat scores: Most-disturbed site imposes uniform energetic deficits

Reduced fat accumulation, exhibited by both satellite and core species in the most-disturbed site, can also be indicative of an energy deficit. However, three major factors dictate general patterns in fat storage levels. (1) If food resources are abundant enough to allow for strategic fat regulation, then birds subject to variable access to food or to variable energetic stress will store excess fat compared to birds experiencing lower variance in resource levels or lower variance in energetic stress [96,97]. For example, birds in winter often have higher fat scores than those in fall or spring [98–102]. Similarly, dominant birds that have priority of access to food resources have been shown to carry less fat than subordinates ([99,103,104]; but see [97,105]). (2) Alternatively, resource levels may not be sufficient to allow for a strategic regulation of fat accumulation, in which case animals in poor-quality environments or those subject to stressful conditions are expected to have relatively low fat scores simply because resources are insufficient to allow for the storage of excess energy (see [106]). (3) High predation risk can also cause birds to reduce fat loads in order to increase maneuverability during anti-predator escape behavior [107]. However, under harsh energetic environments, parids in particular have been shown to bias body condition regulation toward minimization of starvation risk [107].

Increased foraging time and larger home range sizes indicate poor foraging quality at the most-disturbed site. Poor foraging quality coupled with relatively low raptor calling rates suggest that the reduced fat scores at the most-disturbed site are best explained by insufficient resources rather than strategic energetic regulation or patterns of predation risk. The relationship between insufficient resources and fat storage has been confirmed both in the laboratory [88] and in the field [101,103,108,109]. Importantly, decreased body condition, along with increased levels of stress hormones (i.e. corticosterone), were previously shown in birds from strongly disturbed sections of our mid-disturbed site compared to body condition and hormone levels in birds collected from our undisturbed site ([70]; also see [108]).

### Fitness implications of an energetic deficit in core species

Evidence of an energetic deficit provides insight into variation in levels of two fitness indices: fledgling counts and survivorship. For instance, an energetic deficit in winter can have detrimental effects on body condition and in turn negatively affect breeding success the following breeding season (see [110]). Indeed, we found that fledgling counts for TUTI decreased with disturbance, such that TUTI fledgling count was lower in the disturbed sites. Although CACH fledgling counts were consistent across the gradient, the observation of only a single CACH family flock at the most disturbed site further reflects the reduced quality of that habitat. These results illustrate that CACH and TUTI, along with other habitat generalists that are capable of living under a broad range of conditions, none-the-less suffer lower breeding success when living in disturbed or secondary-growth habitats [111–113].

CACH and TUTI biannual survival rates were also lower at the most-disturbed site relative to the mid-disturbed site. These results are likely related to the reduced quality of foraging habitat at the most-disturbed site [97,106,114]. Reduced survival could also be linked back to the reproductive costs experienced in low quality foraging habitat, where an energetic deficit increases the cost of reproduction (see [110]).

It is important to note, however, that the estimates for TUTI survival rates were lowest at the undisturbed site. The unexpectedly low TUTI survival estimates at the undisturbed site could potentially be explained by higher adult emigration from this site than from the others. Indeed, the higher fledging rates could increase demographic pressure and, in conjunction with high quality foraging habitat, cause heightened competition for territories leading to greater turnover rates via adult death or emigration [115,116].

### Flocking propensity by satellites extends into the breeding season in poor-quality foraging habitat

Mixed-species flock organization (i.e. flock size, stability, and species richness and composition) typically changes across disturbance gradients [8,23,25,27,32–35]. In our study, however, the same flocking species (CACH, TUTI, WBNU, DOWO) were present across the disturbance gradient regardless of the differences in forest structure among our sites. This is likely because our study species are relatively disturbance-tolerant and fairly common [42,43] in contrast to the more disturbance-sensitive flocking species typically studied [25]. The size of our mixed-species flocks was also unaffected by disturbance despite variability in core species abundance among sites; this too contrasts with previous studies that report a relationship between the abundance of flocking species and number of members in a flock (e.g. [27,117]). The source of this discrepancy is unclear, but it is possible that we would have found an abundance/flock-composition effect had the differences in abundance estimates between our sites been more pronounced.

Importantly, the number of satellite members remained high in the breeding season at the most-disturbed site, whereas the number of satellite members decreased during the breeding season as expected in the less disturbed sites. We also found that the core species appear to be equally attractive to the satellite species independent of site. Our results suggest that the impact of disturbance on multiple behavioral and life history traits in the core species does not diminish satellite attraction to them. The fact that the mixed-species flocks extend into the breeding season in the most disturbed sites underscores the importance of these associations in poor-quality habitats. Of course, further research is needed to determine whether the combination of habitat disturbance effects on core species and habitat degradation also makes the information transfer from core to satellite species (see [15,16,44]) more salient than in higher quality foraging habitat.

In contrast, the disturbance effects experienced by core and satellite species did not affect interspecific association patterns. CACH avoidance of TUTI was uniform across our disturbance gradient. Lower food availability is known to exacerbate interspecific competition between TUTI and CACH [118,119] and other species in the family Paridae [120–122]; therefore, we expected some shift in their attraction to each other across our sites. A consistent aversion to TUTI could be attributed to the fact that CACH survivorship tends to decrease in the presence of TUTI irrespective of habitat quality [119]. Indeed, we found that survivorship of chickadees was lower than the more socially dominant titmice across sites, and the discrepancy in survival rates was largest during the nonbreeding season when food is scarcer, and intra- and inter-specific competition is resultantly stronger [123]. Cimprich and Grubb ([118]) also found that CACH tend to be energetically stressed in the presence of TUTI, to whom they are normally subordinate, and subordinate species in parid flocks are generally forced to use lower quality microhabitats [120–122]. This conclusion is further supported by our results that CACH spent a greater fraction of their time budget on foraging compared to TUTI. Interestingly, the aversion of CACH to TUTI was moderated by the presence of satellite species. This could result if the intensity of the dominance relationships between TUTI and CACH is diminished by the presence of other species.

### Linking effects to disturbed forest structure

Disturbance effects have been linked to forest vegetative structure, habitat patch size and pattern of forest fragmentation (e.g. [24,31]). All of our sites were embedded in the same second-growth remnant forest network (precluding ‘fragmentation’ effects), and while canopy cover is important to our study species [38], it was invariant across our sites. Therefore, the disturbance effects we detected were likely derived from variation in relative importance of tree species and associated impacts on energetic deficits. CACH and TUTI infrequently utilize walnut trees for foraging, so our two sites dominated by hardwood species other than walnut trees likely provided higher quality foraging habitat [124,125] than the most-disturbed site (where walnuts were ranked second in importance value). Indeed, the increased foraging time budget, reduced fat loads, and increased home range size suggest a food shortage at the most-disturbed site. The likelihood of a food shortage would be higher in winter when food resources are sparsest [124] and for flocks whose home ranges overlapped with the walnut plantations (where walnut trees grew more densely than those naturally occurring outside the plantation stands).

### Conclusion

Our study reveals mechanisms underlying flock composition of birds surviving in remnant forest and links the mechanisms to degradation of foraging habitat. Specifically, our data for core species show that foraging time budgets and home range size increase and fitness indices are negatively impacted in our most-disturbed site. Furthermore, low fat scores indicate an energetic deficit in both core and satellite species in our most-disturbed site. The fact that satellites continued to flock with core species during the breeding season suggests foraging niche expansion that results from mixed-species flocking is particularly important in disturbed sites even beyond the winter season. We therefore conclude that the value of flocking with core species is likely higher for satellite species in poor-quality foraging habitat [126], which underscores the importance of prioritizing the conservation of core species.

The successful conservation of core species and interspecific interactions that maintain animal community integrity in remnant forests will enhance and protect the biodiversity-holding capacity of fragmented landscapes [14,28]. Our study offers several insights of relevance to future conservation efforts. For instance, our finding that core species increased home range size in response to the disturbance-related reduction in foraging habitat quality [25] underscores the importance of considering the effect that disturbance can have on foraging habitat quality and space-use requirements—especially in isolated forest fragments where home range expansion is limited by the size of the fragment. Our results also demonstrate that even relatively disturbance-tolerant flocking species can benefit from a small, stand-scale management perspective that minimizes disturbance to the vegetative integrity within forest remnants (corridors, patches, etc.). Finally, our use of more refined metrics (e.g. space use and foraging behavior rather than species richness) provided key insight into the impact potential of forest disturbance on low-diversity mixed-species flocking systems.

## Supporting information

S1 Appendix Passive acoustic recording methods. (DOCX)

S2 Appendix Acoustic scan sampling. (DOCX)

S1 Fig Aerial image of our three sites, each at a different stand of a forest remnant network. Stephens Forest (most-disturbed site) is approximately 65 km from Ross Biological Reserve (undisturbed site). Ross Biological Reserve and Martell Forest (mid-disturbed site) are separated by approximately 5 km.
(TIFF)

S1 Table Change in tree population measures following timber harvest. There was a small decrease in mean measures of basal area, population density (pop. density), and total basal area (cover) from pre-harvest to post-harvest at the timber-harvest site. Lower and upper confidence limits and standard errors (SE) are listed for each variable, as well as standard deviation, and minimum and maximum values of each variable.
(DOCX)

S2 Table Sample summary for banded Carolina chickadees and tufted titmice among the three sites in West-central Indiana. (DOCX)

S3 Table Candidate model set for survivorship encounter data. If parameters were non-estimable with link function, a sin function was applied instead. Apparent survival probability is denoted as *Φ*, probability of detection is denoted as *p*, and estimates of each were averaged across candidate models with a cumulative weight of ≤ 0.95. Parameters were modeled as group (‘study site’), time on a biannual (‘Survey period’) or seasonal (‘season’) basis, or constant (.). ‘-1’ indicates exclusion of intercept.
(DOCX)

S4 Table QAIC model set for abundance analysis. Covariate models on intercept (α) and individual heterogeneity (σ) parameters include intercept only (.) and group effect (g), while the number of unmarked individuals in the population (U) included group effect (g).
(DOCX)

S5 Table Relative frequencies, relative dominances, relative densities, and importance values of trees (≥ 10 cm dbh) at the mid-disturbed site (MART), most-disturbed site (STEP), and the undisturbed site (ROSS). (DOCX)

S6 Table Cormack-Jolly-Seber (CJS) model selection results for CACH and TUTI captured in Indiana from fall 2015 to fall 2017. Models with cumulative weight of ≤ 0.95 are shown.
(DOCX)

S7 Table Model-averaged detection probability estimates (± SE)^a^ for each site. (DOCX)
