# Supplementary material for: Flocking propensity by satellites, but not core members of mixed-species flocks, increases when individuals experience energetic deficits in a poor-quality foraging habitat
Source: PLoS One. 2019 Jan 9;14(1):e0209680. doi: 10.1371/journal.pone.0209680 (PMC6326460; doi:10.1371/journal.pone.0209680)
Supplement: S1 Appendix — (DOCX) [file pone.0209680.s001.docx]

**Passive acoustic recording**

We conducted passive acoustic recordings using Wildlife Acoustics SM3 song meters (Wildlife Acoustics Inc., Massachusetts, USA). A total of ten SM3 units were rotated between the three sites throughout 2016, such that five SM3 units recorded for thirty minutes around the time of true dawn (ranging from -77 minutes +72 minutes to time since sunrise) for every month at each site (for a total of 1218 recording hours). Sampling effort varied within and across sites throughout the year (see table on next page) due to unexpected incidences of deployment or battery failure (a total of 742, 965, and 729 audio files were recorded at the undisturbed, mid-disturbed, and most-disturbed site, respectively).

The SM3 units were mounted on trees approximately 2 m from the ground and were separated from one another by at least 100 m within each site. Daily audio files were saved to internal memory as 16 bit, at a sampling rate of 32 kHz at 24.0 gain. The SM3 units were set to record WAV files on stereo channels using foam windscreen-covered SMM-A1 microphones, which are weatherproof, omnidirectional and have a flat (+/-10 dB) frequency response up to 20 kHz and a sensitivity of -11 ± 4 dB (0 dB = 1V/pa at 1 KHz), with a signal-to-noise ratio of > 68 dB (Wildlife Acoustics 2014). An internal microphone was attached to the SM3 unit on the left channel, while an external microphone was connected to the right channel via an extension cable. The external microphone was hung facing downward from a thin tree sapling (< 10 cm dia), 2 m from the ground, and 40 m away from the SM3 unit. Each of the external microphones were separated from one another by approximately 130 m.

| **Site** | **Number of 0.5 hr dawn recording files per SM3 Unit ID for every month at each site** | | | | | **Total 0.5 hr dawn recording files per month** |
| --- | --- | --- | --- | --- | --- | --- |
|  | **1** | **2** | **3** | **4** | **5** |  |
| **January (0700 hr)** | | | | | | |
| Undisturbed | 5 | 5 | 5 | 5 | 5 | 25 |
| Mid-disturbed | 8 | 8 | 8 | 8 | 8 | 40 |
| Most-disturbed | 14 | 5 | 14 | 14 | 14 | 61 |
| **February (0700 hr)** | | | | | | |
| Undisturbed | 9 | 9 | 9 | 9 | 9 | 45 |
| Mid-disturbed | 20 | 20 | 20 | 20 | 17 | 97 |
| Most-disturbed | 16 | 17 | 17 | 5 | 5 | 60 |
| **March (0600 hr)** | | | | | | |
| Undisturbed | 14 | 14 | 14 | 14 | 13 | 69 |
| Mid-disturbed | 14 | 15 | 15 | 15 | 15 | 74 |
| Most-disturbed | 5 | 5 | 5 | 5 | 5 | 25 |
| **April (0600 hr)** | | | | | | |
| Undisturbed | 13 | 13 | 13 | 13 | 13 | 65 |
| Mid-disturbed | 25 | 25 | 25 | 25 | 25 | 125 |
| Most-disturbed | 9 | 9 | 9 | 9 | 9 | 45 |
| **May (0600 hr)** | | | | | | |
| Undisturbed | 14 | 16 | 17 | 16 | 12 | 75 |
| Mid-disturbed | 11 | 7 | 12 | 13 | 9 | 52 |
| Most-disturbed | 13 | 13 | 13 | 13 | 13 | 65 |
| **June (0600 hr)** | | | | | | |
| Undisturbed | 13 | 7 | 13 | 13 | 13 | 59 |
| Mid-disturbed | 5 | 2 | 4 | 6 | 6 | 23 |
| Most-disturbed | 14 | 12 | 14 | 15 | 10 | 65 |
| **July (0600 hr)** | | | | | | |
| Undisturbed | NA | 12 | 12 | 7 | 7 | 38 |
| Mid-disturbed | 17 | 17 | 17 | 18 | 9 | 78 |
| Most-disturbed | 13 | 13 | 14 | 14 | 13 | 67 |
| **August (0600 hr)** | | | | | | |
| Undisturbed | 9 | 11 | 6 | 9 | 9 | 44 |
| Mid-disturbed | 7 | 5 | 6 | 8 | 6 | 32 |
| Most-disturbed | 12 | 12 | 12 | 6 | 8 | 50 |
| **September (0600 hr)** | | | | | | |
| Undisturbed | 10 | 10 | 4 | 10 | 10 | 44 |
| Mid-disturbed | 13 | 14 | 14 | 7 | 10 | 58 |
| Most-disturbed | 16 | 12 | 16 | 18 | 15 | 77 |
| **October (0600 hr)** | | | | | | |
| Undisturbed | 8 | 9 | 7 | 2 | 10 | 36 |
| Mid-disturbed | 17 | 15 | 16 | 19 | 16 | 83 |
| Most-disturbed | NA | NA | NA | NA | NA | NA |
| **November (0700 hr)** | | | | | | |
| Undisturbed | 23 | 22 | 22 | 23 | 23 | 113 |
| Mid-disturbed | 16 | 16 | 17 | 18 | 10 | 77 |
| Most-disturbed | 3 | 3 | 3 | 3 | 3 | 15 |
| **December (0800 hr)** | | | | | | |
| Undisturbed | 12 | 9 | 10 | 12 | 10 | 53 |
| Mid-disturbed | 15 | 15 | 15 | 15 | 10 | 70 |
| Most-disturbed | 13 | 13 | 13 | 13 | 13 | 65 |
