# Supplementary material for: Flocking propensity by satellites, but not core members of mixed-species flocks, increases when individuals experience energetic deficits in a poor-quality foraging habitat
Source: PLoS One. 2019 Jan 9;14(1):e0209680. doi: 10.1371/journal.pone.0209680 (PMC6326460; doi:10.1371/journal.pone.0209680)
Supplement: S2 Appendix — (DOCX) [file pone.0209680.s002.docx]

**Acoustic Scan Sampling**

For each month of recordings, the Cluster Analysis feature of Kaleidoscope Pro 4.1.0a (Wildlife Acoustics, Maynard, Massachusetts) was used to automatically detect low frequency acoustic signals, including avian predator vocalizations, that happened to be recorded at each site. The clustering algorithm was set to automatically detect and sort similar acoustic signals into clusters based on the following signal parameter settings: 100 – 1000 Hz, 0.1–7.5s duration, 0.35s maximum inter-syllable group. The FFT window was set to 5.33 ms (512 samples at 24 – 48 kHz). The maximum number of states for the target size of the Hidden Markov Model was set to 12, the maximum Euclidean distance to cluster center for building clusters was set to 0.5, and maximum Euclidean distance from cluster center to include in cluster file outputs was set to 1.0.

The author KG manually scanned the cluster content for acoustic detections of common, forest-dwelling avian predators of Indiana, including: Barred owls (*Strix varia*) (BWOD), Eastern screech owl (*Megascops asio*) (EASO), Great-horned owl (*Bubo virginianus*) (GHOW), Red-shouldered hawk (*Buteo jamaicensis*) (RSHA), Cooper’s hawk (*Accipiter cooperii*) (COHA), and Sharp-shinned hawk (*Accipiter striatus*) (SSHA). Using the Kaleidoscope Viewer feature, the portion of the audio file containing each detection was transformed to the frequency domain and displayed as a spectrogram along a frequency range between 0 – 15 kHz (Nyquist frequency) on the y-axis and the duration of the detection in seconds on the x-axis using a Hanning window with 50% overlap. Manual scans were conducted through visual and auditory inspection of the spectrogram content. Although the cluster content contained thousands of low frequency signal detections per month, very few detections were of avian predator vocalizations. For this reason, it was necessary to manually review each detection.
