# Supplementary figures and images for: Flocking propensity by satellites, but not core members of mixed-species flocks, increases when individuals experience energetic deficits in a poor-quality foraging habitat
Source: PLoS One. 2019 Jan 9;14(1):e0209680. doi: 10.1371/journal.pone.0209680 (PMC6326460; doi:10.1371/journal.pone.0209680)

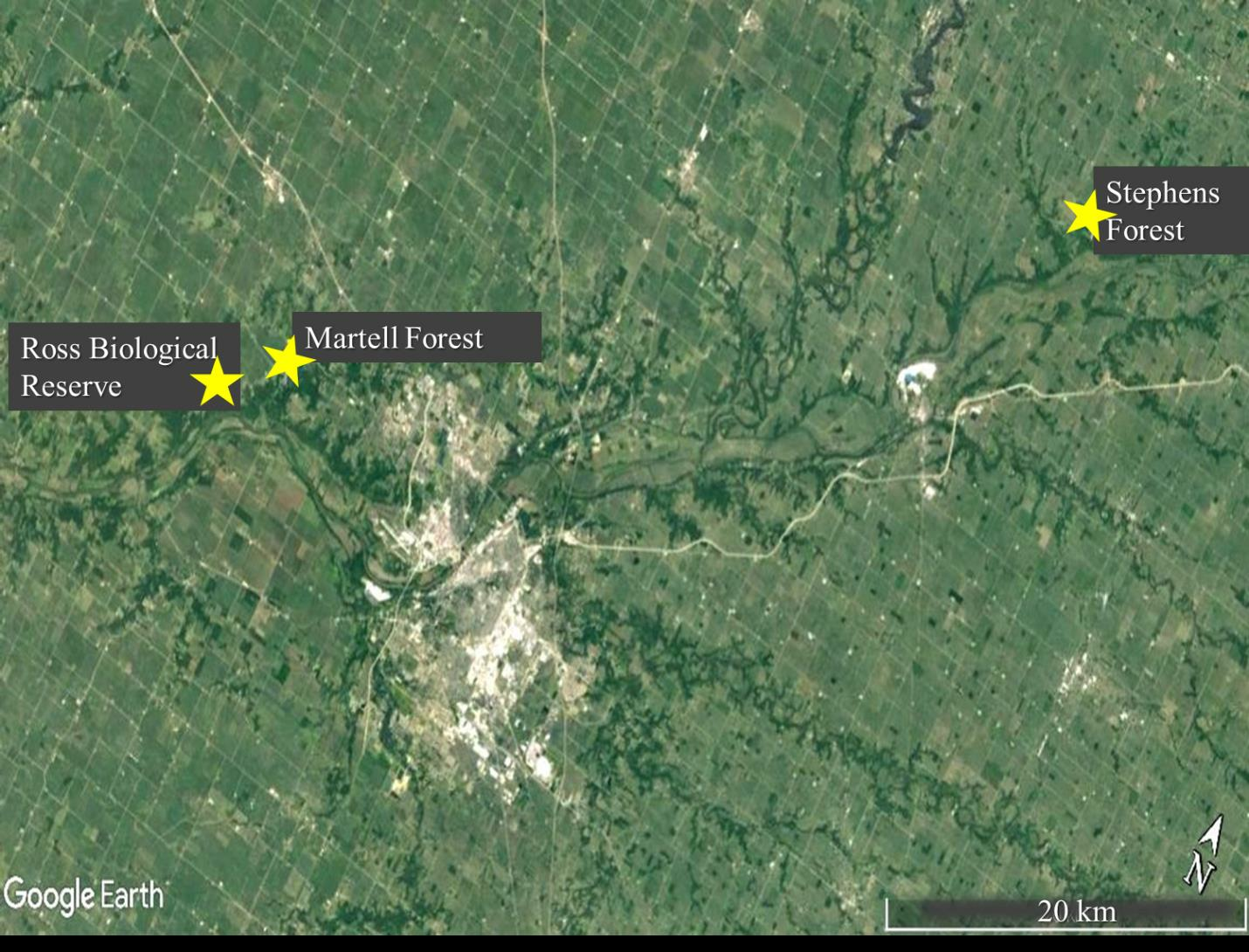

Supplement: S1 Fig — Stephens Forest (most-disturbed site) is approximately 65 km from Ross Biological Reserve (undisturbed site). Ross Biological Reserve and Martell Forest (mid-disturbed site) are separated by approximately 5 km. (TIFF) [file pone.0209680.s003.tiff]
