# Supplementary material for: Flocking propensity by satellites, but not core members of mixed-species flocks, increases when individuals experience energetic deficits in a poor-quality foraging habitat
Source: PLoS One. 2019 Jan 9;14(1):e0209680. doi: 10.1371/journal.pone.0209680 (PMC6326460; doi:10.1371/journal.pone.0209680)
Supplement: S1 Table — There was a small decrease in mean measures of basal area, population density (pop. density), and total basal area (cover) from pre-harvest to post-harvest at the timber-harvest site. Lower and upper confidence limits and standard errors (SE) are listed for each variable, as well as standard deviation, and minimum and maximum values of each variable. (DOCX) [file pone.0209680.s004.docx]

**S1 Table Change in tree population measures following timber harvest**. Lower and upper confidence limits (± SE) are listed for each variable, as well as the standard deviation and minimum and maximum.

| **Variable** | **N** | **Mean** | **Lower 95%** | **Upper 95%** | **SE** |
| --- | --- | --- | --- | --- | --- |
| \| **Δ basal area** \| \| --- \| \| **Δ pop. density** \| \| **Δ cover** \| | \| 5 \| \| --- \| \| 5 \| \| 5 \| | \| -0.07 \| \| --- \| \| -0.15 \| \| -0.21 \| | \| -0.27 \| \| --- \| \| -0.32 \| \| -0.48 \| | \| 0.13 \| \| --- \| \| 0.01 \| \| 0.06 \| | \| 0.07 \| \| --- \| \| 0.06 \| \| 0.10 \| |
