# Supplementary material for: Flocking propensity by satellites, but not core members of mixed-species flocks, increases when individuals experience energetic deficits in a poor-quality foraging habitat
Source: PLoS One. 2019 Jan 9;14(1):e0209680. doi: 10.1371/journal.pone.0209680 (PMC6326460; doi:10.1371/journal.pone.0209680)
Supplement: S2 Table — (DOCX) [file pone.0209680.s005.docx]

**S2 Table. Sample summary for banded Carolina chickadees and tufted titmice among the three sites in West-central Indiana.**

| **CACH** | | | | | |
| --- | --- | --- | --- | --- | --- |
| **Excluded from survivorship analysis** | | **Included in survivorship analysis** | | | **Total Banded** |
| **# banded as HY^a^; not seen as adults** | **# banded as adults; not seen post-banding** | **Total** | **Males/Total** | **Females/Total** |  |
| ***Undisturbed site*** | | | | | |
| 49 | 12 | 17 | 0.71 | 0.29 | 78 |
| ***Intermediate disturbed site*** | | | | | |
| 20 | 3 | 52 | 0.60 | 0.40 | 75 |
| ***Most-disturbed site*** | | | | | |
| 18 | 6 | 23 | 0.52 | 0.48 | 47 |
| **Total** | | | | | |
| 87 | 21 | 92 | 0.60 | 0.40 | 200 |
| **TUTI** | | | | | |
| **Excluded from survivorship analysis** | | **Included in survivorship analysis** | | | **Total Banded** |
| **# banded as HY; not seen as adults** | **# banded as adults; not seen post-banding** | **Total** | **Males/Total** | **Females/Total** |  |
| ***Undisturbed site*** | | | | | |
| 13 | 19 | 44 | 0.50 | 0.50 | 76 |
| ***Intermediate disturbed site*** | | | | | |
| 10 | 17 | 42 | 0.50 | 0.50 | 69 |
| ***Most-disturbed site*** | | | | | |
| 8 | 14 | 32 | 0.53 | 0.47 | 54 |
| **Total** | | | | | |
| 31 | 50 | 118 | 0.51 | 0.49 | 199 |

^a^ ‘HY’ refers to birds in the year they hatched.
