# Supplementary material for: Flocking propensity by satellites, but not core members of mixed-species flocks, increases when individuals experience energetic deficits in a poor-quality foraging habitat
Source: PLoS One. 2019 Jan 9;14(1):e0209680. doi: 10.1371/journal.pone.0209680 (PMC6326460; doi:10.1371/journal.pone.0209680)
Supplement: S3 Table — If parameters were non-estimable with link function, a sin function was applied instead. Apparent survival probability is denoted as Φ, probability of detection is denoted as p, and estimates of each were averaged across candidate models with a cumulative weight of ≤ 0.95. Parameters were modeled as group (‘study site’), time on a biannual (‘Survey period’) or seasonal (‘season’) basis, or constant (.). ‘-1’ indicates exclusion of intercept. (DOCX) [file pone.0209680.s006.docx]

**S3 Table Candidate model set for survivorship encounter data.** Apparent survival probability is denoted as *Ф,* probability of detection is denoted as *p*, and estimates of each were averaged across candidate models with a cumulative weight of ≤ 0.95. Parameters were modeled as group (‘study site’), time on a biannual (‘Survey period’) or seasonal (‘season’) basis, or constant (.). ‘-1’ indicates exclusion of intercept.

| **Structure ID** | **Apparent Survival (Ф)** | **Detection probability (p)** |
| --- | --- | --- |
| 1 | Season x study site | Survey period + study site |
| 2 | Study site | Survey period + study site + (survey period x study site) |
| 3 | -1 + study site | -1 + survey period |
| 4 | -1 + study site | -1 + study site |
| 5 | -1 + study site | Season + site |
| 6 | -1 + study site | Study site x season |
| 7 | Season + study site | -1 + survey period |
| 8 | -1 + study site | . |
| 9 | Season + study site | Season + study site |
| 10 | Season + study site | Survey period + study site |
| 11 | Season + study site | Survey period + study site + (survey period x study site) |
| 12 | Season + study site | Season x study site |
| 13 | Season + study site | Study site |
| 14 | Season + study site | . |
| 15 | Season + study site + (season x study site) | Survey period |
| 16 | Season + study site + (season x study site) | Season + study site |
| 17 | Season + study site + (season x study site) | Season + study site + (season x study site) |
| 18 | Season + study site + (season x study site) | Survey period + study site + (survey period x study site) |
| 19 | Season + study site + (season x study site) | Study site |
| 20 | Season + study site + (season x study site) | . |
| 21 | Study site | Survey period + study site |
