# Supplementary material for: Flocking propensity by satellites, but not core members of mixed-species flocks, increases when individuals experience energetic deficits in a poor-quality foraging habitat
Source: PLoS One. 2019 Jan 9;14(1):e0209680. doi: 10.1371/journal.pone.0209680 (PMC6326460; doi:10.1371/journal.pone.0209680)
Supplement: S4 Table — Covariate models on intercept (α) and individual heterogeneity (σ) parameters include intercept only (.) and group effect (g), while the number of unmarked individuals in the population (U) included group effect (g). (DOCX) [file pone.0209680.s007.docx]

**S4 Table QAIC model set for study site abundance analysis**. Covariate models on intercept (α) and individual heterogeneity (σ) parameters include intercept only (.) and group effect (g), while the number of unmarked individuals in the population (U) included group effect (g).

| **CACH** | | | | | | |
| --- | --- | --- | --- | --- | --- | --- |
| **Model** | **AIC_c_** | **Delta AIC_c_** | **Delta AIC_c_ Weight** | **Model Likelihood** | **K^np^** | **Deviance** |
| **α(.) σ(.) U(.)** | 186.61 | 0.00 | 0.48 | 1.0000 | 3 | 180.04 |
| **α(.) σ(.) U(g)** | 188.73 | 2.12 | 0.16 | 0.3465 | 5 | 177.23 |
| **α(.) σ(g) U(.)** | 188.78 | 2.17 | 0.16 | 0.3375 | 5 | 177.28 |
| **α(g) σ(.) U(.)** | 188.92 | 2.31 | 0.15 | 0.3152 | 5 | 177.42 |
| **α(g) σ(g) U(.)** | 192.45 | 5.84 | 0.03 | 0.0540 | 7 | 175.50 |
| **α(g) σ(.) U(g)** | 192.94 | 6.32 | 0.02 | 0.0424 | 7 | 175.99 |
| **α(.) σ(g) U(g)** | 192.98 | 6.36 | 0.02 | 0.0415 | 7 | 176.03 |
| **TUTI** | | | | | | |
| **Model** | **AIC_c_** | **Delta AIC_c_** | **Delta AIC_c_ Weight** | **Model Likelihood** | **K^np^** | **Deviance** |
| **α(.) σ(g) U(.)** | 106.90 | 0.00 | 0.48 | 1.00 | 4 | 97.74 |
| **α(.) σ(.) U(.)** | 108.39 | 1.48 | 0.23 | 0.48 | 3 | 101.56 |
| **α(.) σ(.) U(g)** | 110.25 | 3.35 | 0.09 | 0.19 | 5 | 98.03 |
| **α(.) σ(g) U(g)** | 110.60 | 3.70 | 0.08 | 0.16 | 6 | 95.37 |
| **α(g) σ(g) U(g)** | 111.10 | 4.20 | 0.06 | 0.12 | 7 | 92.62 |
| **α(g) σ(g) U(.)** | 112.16 | 5.26 | 0.03 | 0.07 | 6 | 96.93 |
| **α(g) σ(.) U(.)** | 112.42 | 5.52 | 0.03 | 0.06 | 5 | 100.20 |
| **α(g) σ(.) U(g)** | 114.53 | 7.63 | 0.01 | 0.02 | 7 | 96.05 |
