# Supplementary material for: Flocking propensity by satellites, but not core members of mixed-species flocks, increases when individuals experience energetic deficits in a poor-quality foraging habitat
Source: PLoS One. 2019 Jan 9;14(1):e0209680. doi: 10.1371/journal.pone.0209680 (PMC6326460; doi:10.1371/journal.pone.0209680)
Supplement: S5 Table — (DOCX) [file pone.0209680.s008.docx]

**S5 Table. Relative frequencies, relative dominances, relative densities, and importance values of trees (≥ 10 cm dbh) at the mid-disturbed site (MART), most-disturbed site (STEP), and the undisturbed site (ROSS).**

| **Study Site** | **Tree Genus** | **Relative Frequency** | **Relative Dominance** | **Relative Density** | **Importance Value** |
| --- | --- | --- | --- | --- | --- |
| MART | Magnolia | 0.00 | 0.00 | 0.00 | 0.00 |
| MART | Ironwood | 0.01 | 0.00 | 0.01 | 0.02 |
| MART | Walnut | 0.01 | 0.01 | 0.01 | 0.03 |
| MART | Buckeye | 0.01 | 0.01 | 0.02 | 0.04 |
| MART | Dogwood | 0.02 | 0.01 | 0.02 | 0.05 |
| MART | Ash | 0.02 | 0.02 | 0.02 | 0.06 |
| MART | Hophornbeam | 0.03 | 0.01 | 0.02 | 0.07 |
| MART | Cherry | 0.03 | 0.03 | 0.02 | 0.08 |
| MART | Hackberry | 0.07 | 0.01 | 0.02 | 0.11 |
| MART | Sassafrass | 0.03 | 0.03 | 0.05 | 0.11 |
| MART | Poplar | 0.03 | 0.08 | 0.04 | 0.16 |
| MART | Basswood | 0.07 | 0.13 | 0.10 | 0.31 |
| MART | Hickory | 0.12 | 0.12 | 0.12 | 0.36 |
| MART | Elm | 0.13 | 0.08 | 0.16 | 0.36 |
| MART | Maple | 0.22 | 0.17 | 0.26 | 0.65 |
| MART | Oak | 0.20 | 0.39 | 0.21 | 0.80 |
| ROSS | Mulberry | 0.00 | 0.00 | 0.00 | 0.00 |
| ROSS | Redbud | 0.00 | 0.00 | 0.00 | 0.00 |
| ROSS | Locust | 0.00 | 0.00 | 0.00 | 0.01 |
| ROSS | Sassafrass | 0.00 | 0.00 | 0.00 | 0.01 |
| ROSS | Hophornbeam | 0.01 | 0.00 | 0.01 | 0.02 |
| ROSS | Ironwood | 0.01 | 0.01 | 0.01 | 0.03 |
| ROSS | Sycamore | 0.01 | 0.01 | 0.01 | 0.03 |
| ROSS | Beech | 0.01 | 0.01 | 0.02 | 0.04 |
| ROSS | Basswood | 0.02 | 0.03 | 0.02 | 0.06 |
| ROSS | Hickory | 0.03 | 0.04 | 0.04 | 0.11 |
| ROSS | Cherry | 0.04 | 0.03 | 0.05 | 0.12 |
| ROSS | Walnut | 0.05 | 0.04 | 0.04 | 0.13 |
| ROSS | Ash | 0.06 | 0.07 | 0.07 | 0.19 |
| ROSS | Hackberry | 0.07 | 0.05 | 0.10 | 0.22 |
| ROSS | Elm | 0.15 | 0.07 | 0.13 | 0.36 |
| ROSS | Oak | 0.15 | 0.17 | 0.12 | 0.44 |
| ROSS | Maple | 0.20 | 0.12 | 0.20 | 0.53 |
| ROSS | Poplar | 0.17 | 0.36 | 0.21 | 0.73 |
| STEP | Sweetgum | 0.00 | 0.00 | 0.00 | 0.00 |
| STEP | Sycamore | 0.00 | 0.00 | 0.00 | 0.00 |
| STEP | Mulberry | 0.00 | 0.00 | 0.00 | 0.00 |
| STEP | Hophornbeam | 0.01 | 0.00 | 0.00 | 0.01 |
| STEP | Basswood | 0.00 | 0.01 | 0.01 | 0.01 |
| STEP | Hackberry | 0.01 | 0.00 | 0.00 | 0.02 |
| STEP | Hickory | 0.01 | 0.01 | 0.01 | 0.04 |
| STEP | Beech | 0.02 | 0.01 | 0.01 | 0.05 |
| STEP | Locust | 0.01 | 0.02 | 0.02 | 0.05 |
| STEP | Cherry | 0.03 | 0.03 | 0.03 | 0.09 |
| STEP | Magnolia | 0.03 | 0.06 | 0.03 | 0.12 |
| STEP | Ash | 0.04 | 0.05 | 0.04 | 0.13 |
| STEP | Oak | 0.06 | 0.07 | 0.05 | 0.18 |
| STEP | Pine | 0.04 | 0.12 | 0.08 | 0.24 |
| STEP | Poplar | 0.07 | 0.14 | 0.09 | 0.30 |
| STEP | Elm | 0.12 | 0.08 | 0.13 | 0.33 |
| STEP | Walnut | 0.11 | 0.14 | 0.12 | 0.37 |
| STEP | Maple | 0.41 | 0.32 | 0.40 | 1.13 |
