# Supplementary material for: Flocking propensity by satellites, but not core members of mixed-species flocks, increases when individuals experience energetic deficits in a poor-quality foraging habitat
Source: PLoS One. 2019 Jan 9;14(1):e0209680. doi: 10.1371/journal.pone.0209680 (PMC6326460; doi:10.1371/journal.pone.0209680)
Supplement: S6 Table — Models with cumulative weight of ≤ 0.95 are shown. (DOCX) [file pone.0209680.s009.docx]

**S6 Table Cormack-Jolly-Seber (CJS) model selection results for CACH and TUTI captured in Indiana from fall 2015 to fall 2017.** Models with cumulative weight of ≤ 0.95 are shown.

| **Candidate Model Structure** | **ΔAIC_c_*** | **AIC_c_*** | **Model Weight** | ***K*** | **Deviance** |
| --- | --- | --- | --- | --- | --- |
| **CACH** | | | | | |
| 1 (*Ф* _season: study site_, p _survey period + study site_) | 0.00 | 232.54 | 0.34 | 8 | 38.28 |
| 10 (*Ф* _season + study site_, p _survey period + study site_) | 0.55 | 233.08 | 0.26 | 8 | 38.82 |
| 21 (*Ф* _study site_, p _survey period + study site_) | 1.55 | 234.09 | 0.16 | 7 | 42.08 |
| 3 (*Ф*_-1 + study site_, p_-1 + survey period_) | 3.60 | 236.14 | 0.06 | 6 | 46.34 |
| 7 (*Ф* _season + study site_, p_-1 + survey period_) | 4.00 | 236.53 | 0.05 | 7 | 44.52 |
| 15 (*Ф* _season * study site_, p _survey period_) | 4.05 | 236.59 | 0.04 | 8 | 42.33 |
| 11 (*Ф* _season + study site_, p _survey period * study site_) | 4.37 | 236.91 | 0.04 | 11 | 35.69 |
|  | | | | | |
| **TUTI** | | | | | |
| 7 (*Ф* _season + study site_, p_-1 + survey period_) | 0.00 | 233.36 | 0.45 | 8 | 35.47 |
| 3 (*Ф* _-1 + study site_, p_-1 + survey period_) | 1.71 | 235.07 | 0.19 | 7 | 39.36 |
| 1 (*Ф* _season : study site_, p _survey period + study site_) | 2.99 | 236.36 | 0.10 | 10 | 34.04 |
| 21 (*Ф* _study site_, p _survey period + study site_) | 3.23 | 236.60 | 0.09 | 9 | 36.50 |
| 10 (*Ф* _season + study site_, p _survey period + study site_) | 3.27 | 236.64 | 0.09 | 10 | 34.32 |

*TUTI AICc values are corrected for quasilikelihood overdispersion (QAICc) of 1.345
