# Supplementary material for: Flocking propensity by satellites, but not core members of mixed-species flocks, increases when individuals experience energetic deficits in a poor-quality foraging habitat
Source: PLoS One. 2019 Jan 9;14(1):e0209680. doi: 10.1371/journal.pone.0209680 (PMC6326460; doi:10.1371/journal.pone.0209680)
Supplement: S7 Table — (DOCX) [file pone.0209680.s010.docx]

**S7 Table. Model-averaged detection probability estimates (± SE)^a^ for each site.**

| **Survey Period**^b^ | **CACH** | | **TUTI** | | |
| --- | --- | --- | --- | --- | --- |
|  | Mid-disturbed site | Most-disturbed site | Undisturbed site | Mid-disturbed site | Most-disturbed site |
| 2/15-6/1 (2016) | 0.67 ± 0.14 | 0.86 ± 0.10 | 0.72 ± 0.13 | 0.69 ± 0.12 | 0.68 ± 0.14 |
| 9/1-12/1 (2016) | 0.71 ± 0.11 | 0.89 ± 0.08 | 0.36 ± 0.12 | 0.32 ± 0.09 | 0.31 ± 0.09 |
| 2/15-6/1 (2017) | 0.69 ± 0.16 | 0.88 ± 0.11 | 0.93 ± 0.27 | 0.92 ± 0.30 | 0.92 ± 0.32 |
| 9/1-12/1 (2017) | 0.24 ± 0.08 | 0.51 ± 0.19 | 0.28 ± 0.11 | 0.25 ± 0.08 | 0.24 ± 0.08 |

^a^ Model-averaged estimates are from models with cumulative weight ≤ 0.95.

^b^ There is no detection probability for the first survey period (9/1-12/1 of 2015).
